# Neddylation of RhoA impairs its protein degradation and promotes renal interstitial fibrosis progression in diabetic nephropathy

**DOI:** 10.1038/s41401-024-01460-z

**Published:** 2025-02-03

**Authors:** Xue-qi Li, Bo Jin, Si-xiu Liu, Yan Zhu, Nan Li, Qing-yan Zhang, Cheng Wan, Yuan Feng, Yue-xian Xing, Kun-ling Ma, Jing Liu, Chun-ming Jiang, Jian Lu

**Affiliations:** 1 Institute of Nephrology, Nanjing Drum Tower Hospital, School of Medicine, Southeast University, Nanjing, 210008 China; 2 Department of Nephrology, Nanjing Drum Tower Hospital, The Affiliated Hospital of Nanjing University Medical School, Nanjing, 210008 China; 3 Department of Endocrinology, The Third Affiliated Hospital of Soochow University, Changzhou, 213000 China; 4 Department of Nephrology, The Second Affiliated Hospital, Zhejiang University, School of Medicine, Hangzhou, 310009 China

**Keywords:** neddylation, RhoA, renal fibrosis, diabetic nephropathy

## Abstract

Diabetic nephropathy (DN) is a common and serious complication of diabetes, characterized by chronic fibro-inflammatory processes with an unclear pathogenesis. Renal fibrosis plays a significant role in the development and progression of DN. While recent research suggests that the neddylation pathway may influence fibrotic processes, its specific dysregulation in DN and the underlying mechanisms remain largely unexplored. This study identified the neddylation of RhoA as a novel post-translational modification that regulates its expression and promotes renal fibrosis in DN. We here demonstrated that two key components of the neddylation pathway—NEDD8-activating enzyme E1 subunit 1 (NAE1) and NEDD8—are significantly upregulated in human chronic kidney disease (CKD) specimens compared to healthy kidneys, implicating neddylation in CKD-associated fibrosis. Our findings further revealed that both pharmacological inhibition of neddylation using MLN4924 and genetic knockdown of NAE1 mitigate renal fibrosis in mouse models of streptozotocin-induced diabetes and unilateral ureteral obstruction (UUO). Immunoprecipitation-mass spectrometry (IP-MS) and subsequent function assays demonstrated a direct interaction between RhoA and NEDD8. Importantly, neddylation inhibition reduced RhoA protein expression, highlighting a potential therapeutic target. Additionally, a positive correlation was noted between elevated NEDD8 mRNA levels and RhoA mRNA expression in human CKD specimens. RhoA overexpression counteracted the antifibrotic effects of neddylation inhibition, underscoring its critical role in fibrosis progression. Mechanistically, we unveiled that neddylation enhances RhoA protein stability by inhibiting its ubiquitination-mediated degradation, which subsequently activates the ERK1/2 pathway. Collectively, this study provides novel insights into NAE1-dependent RhoA neddylation as a key contributor to renal fibrosis in DN.

**Figure:**
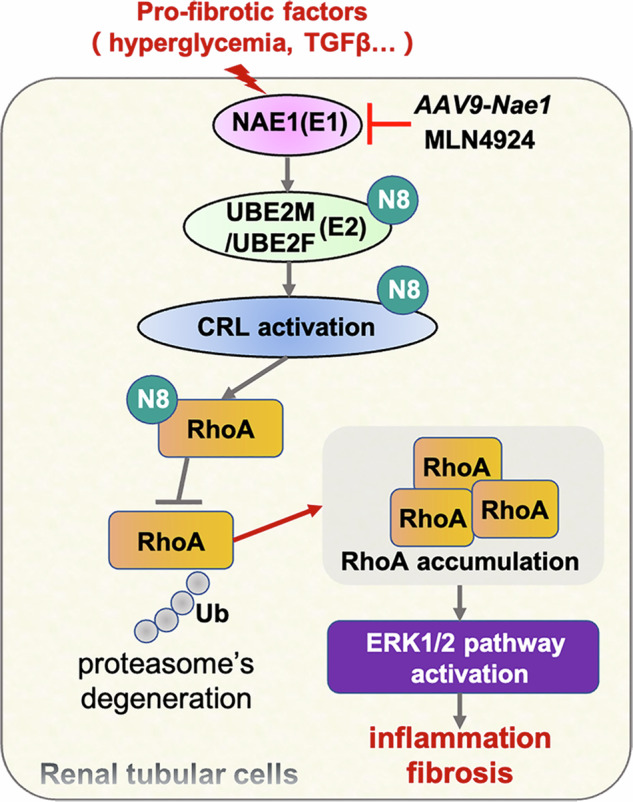
The NAE1 protein mediates RhoA protein hyper-neddylation and subsequent stabilization of the RhoA protein, which, in turn, contributes to the development of renal fibrosis and inflammation through an ERK1/2-dependent mechanism. Consequently, targeting neddylation inhibition represents a viable therapeutic approach for the treatment of renal fibrosis in DN.

The NAE1 protein mediates RhoA protein hyper-neddylation and subsequent stabilization of the RhoA protein, which, in turn, contributes to the development of renal fibrosis and inflammation through an ERK1/2-dependent mechanism. Consequently, targeting neddylation inhibition represents a viable therapeutic approach for the treatment of renal fibrosis in DN.

## Introduction

Diabetic nephropathy (DN) is a global health concern and one of the leading causes of chronic kidney disease (CKD) [1]. It is histopathologically characterized by glomerular basement membrane thickening, mesangial expansion, and tubulointerstitial fibrosis. Emerging evidence highlights the critical role of tubular injury in DN progression, surpassing glomerular pathology as a predictor of CKD [2]. Resident renal tubular epithelial cells (RTECs) play a dual role in renal fibrosis as both victims of injury and initiators of fibrotic processes [3]. Upon damage, RTECs undergo partial epithelial-to-mesenchymal transitions while remaining within tubule basement membranes [4]. This transition is marked by co-expression of epithelial and mesenchymal markers, enabling damaged RTECs to release paracrine signals, such as pro-inflammatory and profibrotic factors, into the interstitial space, thereby fostering a microenvironment conducive to inflammation and fibrosis [5].

Neddylation is a pivotal post-translational modification (PTM) involving the conjugation of the ubiquitin-like protein, neuronal precursor cell-expressed developmentally downregulated protein 8 (NEDD8), to specific target proteins [6, 7]. This process is mediated by a cascade of activities of enzymes, including NEDD8-activating enzyme E1 (comprising NAE1 and UBA3), NEDD8 conjugating enzyme E2 (UBC12), and NEDD8 E3 ligase. Among the well-studied substrates of neddylation are cullin proteins, which serve as scaffolds for cullin-RING E3 ligases (CRLs) [8, 9]. The cullin family, known as scaffold components of CRLs, has been extensively studied as a physiological substrate of neddylation [10]. Numerous studies have highlighted that aberrant activation of neddylation contributes to the progression of various human cancers, including lung cancer, where its hyperactivation is often associated with poor patient survival outcomes [11]. A notable therapeutic strategy involves MLN4924 (pevonedistat), a small-molecule inhibitor targeting NAE1, which inhibits the neddylation E1 enzyme, which has undergone extensive clinical trials for its potential as an anticancer agent [12, 13]. Beyond oncology, recent studies have implicated the neddylation pathway in fibrotic conditions, such as liver fibrosis [14, 15]. Conversely, neddylation inhibition by MLN4924 has shown promise in suppressing inflammation and preventing atherogenesis [16]. Neddylation regulates diverse cellular processes, including transcriptional regulation, cell cycle progression, ribosome biogenesis, inflammatory responses [17], and glucose lipid metabolism [18]. This regulatory control is mediated through the PTM of target proteins, altering their activity, stability, subcellular localization, and DNA binding affinity. Despite these advancements, the role of neddylation in renal fibrosis remains largely unexplored.

To address this gap, our study investigated the role of neddylation in renal fibrosis using a unilateral ureter obstruction (UUO) mouse model and a streptozotocin (STZ)-induced diabetic mouse model. Our findings indicate that neddylation is upregulated in the renal cortex following UUO, with pronounced NEDD8 expression in RTECs. Furthermore, pharmacological inhibition of neddylation or suppression of NAE1 gene expression effectively mitigated renal tubulointerstitial fibrosis and improved renal tubular functions. Mechanistically, our study elucidated that neddylation stabilizes RhoA protein by reducing its ubiquitination-mediated degradation, subsequently leading to activation of the ERK1/2 pathway.

## Materials and methods

### Antibodies

In this study, the following primary antibodies were used: anti-NAE1 antibody (bs-12503R) from Bioss (Beijing, China); anti-NEDD8 (2754), anti-p44/42 MAPK (ERK1/2) (4695), and anti-phospho-p44/42 MAPK (pERK1/2) (4370) antibody from Cell Signaling Technology; anti-F4/80 antibody (ab111101) from Abcam; anti-α-smooth muscle actin (α-SMA, A7248), anti-collagen I (A5786) and anti-RhoA (10749-1-AP) antibodies from Proteintech (Wuhan, China); and anti-E-cadherin (BF0219), anti-phospho-p38 MAPK (Thr180/Tyr182, AF4001), anti-p38 MAPK (AF6456), anti-phospho-JNK1/2/3 (Thr183/Tyr185, AF3318), anti-JNK1/2/3 (AF6318), and anti-collagen I (AF7001), anti-collagen III (BF9208) antibodies from Affinity (Wuhan, China).

### Human kidney biopsy sample collection and ethical approval

For immunostaining studies, kidney samples were collected from patients diagnosed as having renal fibrosis and control individuals at Nanjing Drum Tower Hospital, China. Healthy control samples were derived from non-diseased tissues of renal cell carcinoma patients who had undergone tumor tissue removal surgery. All participants provided written informed consent for the use of their kidney biopsy samples in this study. Ethical approval was granted by the Committee on Research Ethics of Nanjing Drum Tower Hospital (Approval Number: 20180-040-01).

### Diabetes modeling

Diabetes was induced in mice via intraperitoneal injections of streptozotocin (STZ; 50 mg/kg in 0.1 mol/L citrate buffer, pH 4.5) for 5 consecutive days. Control mice received citrate buffer injections. Day 0 was designated as the first day of STZ administration. After 2 weeks, mice with fasting blood glucose levels exceeding 16.7 mmol/L were included in the study. Blood and urine samples were collected 16 weeks after STZ injection to measure serum creatinine and blood glucose levels. Body weights were recorded concurrently with blood sample collection.

### Unilateral ureteral obstruction (UUO) mice model

Male C57BL/6J mice (age: 8–10 weeks) were randomly housed under specific pathogen-free conditions, with constant temperature and humidity. All procedures involving experimental animals adhered to the *NIH Guide for the Care and Use of Laboratory Animals* and were approved by the Committee for Animal Research of Nanjing Drum Tower Hospital (Approval Number: 2022AE01023).

The mice were anesthetized with an intraperitoneal injection of pentobarbital sodium (30 mg/kg). After the abdomen was opened, the left ureter was ligated to induce UUO. The abdominal muscles and skin were sutured in sequence, and the mice were placed on a thermostatic heating plate set at 25 °C until they regained consciousness. Two days after surgery, the experimental mice were intraperitoneally injected with MLN4924 (60 mg/kg) every other day for three doses, while the control group was injected with 10% of 2-hydroxypropyl-β-cyclodextrin as the solvent. Seven days after the UUO procedure, the mice were anesthetized with sodium pentobarbital and euthanized. Kidney tissues were harvested for subsequent analysis.

### AAV9 injection

Adeno-associated virus 9 (AAV9) vectors containing shRNA targeting mouse NAE1 (listed in Supplementary Table 1) and empty control vectors were purchased from WZ Biosciences Inc (Jinan, China). The AAV9 titer after packaging was 8.5 × 10^13^ virus genomes/mL, with a dose of 5 × 10^12^ virus genomes/mouse. Two weeks after diabetes was successfully induced in mice, AAV9 carrying NAE1 shRNA was administered via tail vein injection to knock down NAE1 expression. The mice receiving AAV9 with empty vectors were used as controls. The animals were then randomly divided into four groups: (1) control vector injection; (2) AAV9-NAE1 shRNA injection; (3) diabetic mice with control vector injection; and (4) diabetic mice with AAV9-NAE1 shRNA injection. After 16 weeks, the mice were euthanized, and serum and kidney tissue samples were collected for biochemical and histopathological analyses.

### Histopathological analysis

Paraffin-embedded kidney sections were stained using Periodic acid-Schiff (PAS), hematoxylin-eosin (H&E), and Masson’s trichome staining following standard protocols [19]. The stained sections were examined under a light microscope at 40× magnification, and the images were analyzed using Image J software. The kidney injury index was calculated by determining the percentage of damaged renal tubules showing cell lysis or loss of the brush border in PAS-stained sections. Images were captured using an Olympus BX51 microscope, and three random visual fields per sample were quantified. Fibrosis levels were assessed using Masson-stained sections, also visualized using the Olympus BX51 microscope.

### Immunofluorescence staining

Kidney sections were subjected to dewaxing, antigen retrieval, and permeabilization with 0.1% Triton X-100. After the sections were blocked with 5% BSA for 30 min at room temperature, the sections were incubated overnight at 4 °C with primary antibodies. Secondary antibodies conjugated with fluorescent dyes were applied the following day, and the sections were counterstained with DAPI (4′,6-diamidino-2-phenylindole). Microscopic observations were performed using a confocal scanning microscope.

### Cell culture and treatment

Human proximal tubular epithelial cells (HK-2) were sourced from Cellcook (Guangdong, China) and cultured in Dulbecco’s modified Eagle’s medium (DMEM)/F12 medium supplemented with 10% fetal bovine serum (FBS). HEK293T cells were obtained from the American Type Culture Collection and cultured in DMEM supplemented with 10% (v/v) FBS and 1% penicillin/streptomycin. For specific treatments, the cells were treated with human recombinant TGF-β1 (2 or 4 ng/mL, 100-21C, PeproTech) for the indicated durations. Neddylation was inhibited by treating the cells with MLN4924 [20] (10 μmol/L, HY-70062, MCE). Proteasomal degradation was inhibited by treating the cells with MG132 (2 μmol/L, HY-13259, MCE). To activate RhoA, the cells were treated with Narciclasine (0.1 μmol/L, HY-16563, MCE) [21].

### Transfection of plasmids and siRNA

Human NAE1 siRNA (siNAE1) and control vectors were procured from GenePharma (Shanghai, China). The siRNA (siNAE1) sequences, listed in Supplementary Table 1, were transfected into the cells by using Lipofectamine 2000 following the manufacturer’s protocol. For plasmid transfections, Flag-NEDD8, Flag-RhoA, and HA-ubiquitin plasmids were used alongside appropriate controls. The plasmids, sourced from GeneChem, were transfected at a concentration of 0.5–1 μg/mL into pre-seeded cells in 6-well plates or dishes. The transfection efficiency was assessed after 48 h to confirm suitability for subsequent experiments.

### Real-time PCR

Total RNA was extracted using TRIzol reagent per the manufacturer’s protocol, and cDNA was synthesized for quantitative PCR. Changes in gene expression were quantified relative to those in β-actin, used as an internal control, following established methods [22]. Primers for the target genes are detailed in the Supplementary Materials.

### Western blot analysis

Proteins were extracted from kidney tissues and cultured cells for immunoblotting. β-actin or GAPDH was used as the internal control. Protein expression levels were quantified through densitometric analysis and normalized to internal controls.

### Cycloheximide (CHX) chase assays

To determine the half-life of the RhoA protein, cells were treated with CHX (50 μg/mL) for varying durations. Protein lysates were collected at each time point, and RhoA levels were analyzed through Western blotting to estimate degradation rates [23].

### Co-immunoprecipitation assays

Cells were lysed in co-immunoprecipitation (Co-IP) buffer (Thermo Fisher Scientific, MA, USA) supplemented with protease inhibitor cocktails. Lysates were clarified through centrifugation at 12,000 × *g* for 10 min at 4 °C. The supernatants were collected and incubated overnight at 4 °C with specific primary antibodies in the Co-IP buffer. After a third wash with Co-IP buffer, the protein-antibody complexes were eluted by boiling the beads in 1% (*w*/*v*) SDS sample buffer. The following day, protein A magnetic beads were added to the lysates containing the antigen-antibody complexes, and the mixture was incubated at 4 °C for 2 h. Subsequently, the supernatants were discarded, and the remaining magnetic beads containing the antigen-antibody complexes were retained and suspended for further analysis.

### LC-MS/MS analysis

Proteins immunoprecipitated from the HK-2 cells were reduced with 10 mmol/L dithiothreitol and alkylated with 25 mmol/L ammonium iodide acetate. The proteins were then hydrolyzed with trypsin overnight (8–16 h). After desalting and drying, the hydrolyzed polypeptide samples were desalted and dissolved in a loading buffer containing 0.1% formic acid. Then, the polypeptides were analyzed using an Orbitrap Fusion Lumos mass spectrometer (Thermo Scientific, USA). Tandem mass spectrometry detection was performed in the data-dependent acquisition mode, with the complete scanning resolution set at 60,000 (FWHM) and the mass-to-charge ratio range at *m*/*z* 350–1600. In the HCD fragmentation mode, collision energy was established at 30%. Original data files were retrieved and analyzed using Proteome Discoverer 2.4 software for protein identification and quantification.

### RNA-seq and bioinformatics analyses

Total RNA was extracted from HK-2 cells to prepare sequencing libraries. RNA purification, reverse transcription, library construction, and sequencing were performed at Shanghai Majorbio Bio-pharm Biotechnology Co., Ltd (Shanghai, China) following the manufacturer’s instructions. The samples were sequenced using the NovaSeq X Plus platform. Differential gene expression was assessed using the DEseq2 tool, with genes exhibiting an adjusted *P*-value of <0.05 classified as significantly differentially expressed genes (DEGs). Functional enrichment analysis was performed using KEGG pathways to identify metabolic pathways significantly enriched in the DEGs, applying a Bonferroni-corrected *P*-value threshold of 0.05 compared to the whole-transcriptome background. KEGG pathway analysis was conducted using Python’s SciPy library.

### Statistical analysis

Sample sizes for each experiment are specified in the figure legends. Multiple comparisons were made using the Tukey–Kramer method, while Student’s *t*-test was employed to evaluate significant differences between the two groups. *P* < 0.05 was considered statistically significant. All statistical analyses were conducted using GraphPad Prism Software.

## Results

### Activation of the neddylation pathway in renal tubules of diabetic mouse kidneys and high-glucose-stimulated HK-2 cells

**Fig. 1:**
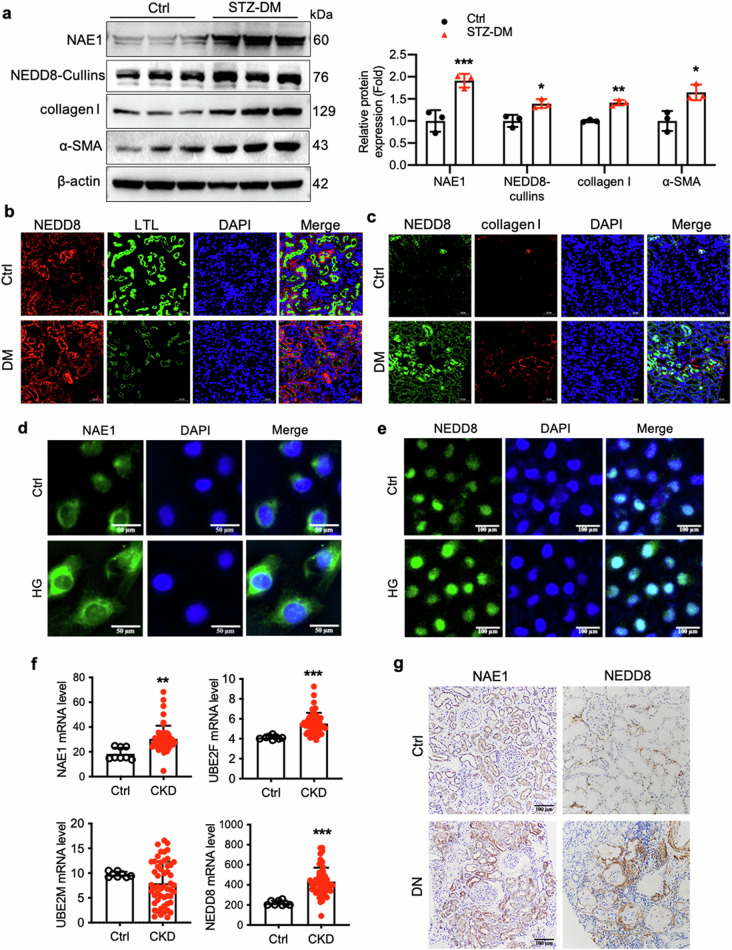
Aberrant expression of neddylation pathway components in proximal tubular epithelial cells (RTECs) during renal fibrosis development. **a** The diabetic mice were induced by streptozotocin (STZ) for 16 weeks. Western blot analysis of collagen I, α-SMA, NAE1, and NEDD8-cullins in cortical kidney tissues of STZ-induced diabetic mice. *n* = 3, mean ± SD, *P < 0.05, **P < 0.01, ***P < 0.001. **b** Representative images of the STZ-induced cortical kidney sections stained with NEDD8 (red), LTL (green), and DNA marker 4', 6-diamidino-2-phenylindole (DAPI, blue). Scale bar = 50 μm. **c** Representative images of STZ-induced cortical kidney sections stained for NEDD8 (green), collagen I (red), and DAPI (blue). Scale bar = 50 μm. **d**, **e** Immunofluorescence staining of NAE1 and NEDD8 in HK-2 cells without or with high-glucose (HG, 30 mmol/L) stimulation. Scale bar= 50 μm or 100 μm. **f** The mRNA levels of NAE1, UBE2M, UBE2F, and NEDD8 in the kidney specimens of CKD (*n* = 53 samples) and control (*n* = 8 samples) in GSE66494 dataset (Probe ID of NAE1: A_23_P77459; Probe ID of NEDD8: A_23_P88420). mean ± SD, **P < 0.01, ***P < 0.001. **g** Immunohistochemistry staining of NEDD8 and NAE1 of the kidney sections from patients with DN or kidney tissues obtained from patients who underwent tumor nephrectomy. Scale bar = 100 μm.

The expression and distribution of NAE1 and NEDD8 in diabetic mouse kidneys have not been previously characterized. Western blot analysis revealed a marked increase in NAE1 and NEDD8 levels in kidneys from STZ-induced mice compared with their age-matched wild-type non-diabetic controls. This was accompanied by elevated renal levels of collagen I and α-SMA, which are markers of fibrosis (Fig. 1a). To further investigate NEDD8 expression in renal tubular epithelial cells, co-immunostaining was performed with NEDD8 and the proximal tubular marker lotus tetragonolobus lectin (LTL) (Fig. 1b). The results indicated a significant upregulation in NEDD8 expression in the proximal renal tubules of diabetic mice. Furthermore, a notable increase in collagen I expression was observed in areas of the renal tubules where NEDD8 levels were high (Fig. 1c). We examined NAE1 and NEDD8 protein expression in high-glucose (HG)-stimulated HK-2 cells through immunofluorescence staining (Fig. 1d, e). Subsequently, transcriptomic data from the Nephroseq database (https://www.nephroseq.org/resource/login.html) were analyzed to investigate NAE1, UBE2M, UBE2F, and NEDD8 expression. These results revealed a significant upregulation of these genes in kidney biopsy tissues from CKD patients in the GSE66494 dataset (*n* = 53 CKD patients, *n* = 8 healthy controls) (Fig. 1f). Moreover, renal biopsies from patients with biopsy-proven DN exhibited elevated NAE1 and NEDD8 expression levels compared with normal kidney tissues obtained from tumor nephrectomy patients without renal disease (Fig. 1g).

### NAE1 and NEDD8 were upregulated in fibrotic kidneys of UUO mice model

**Fig. 2:**
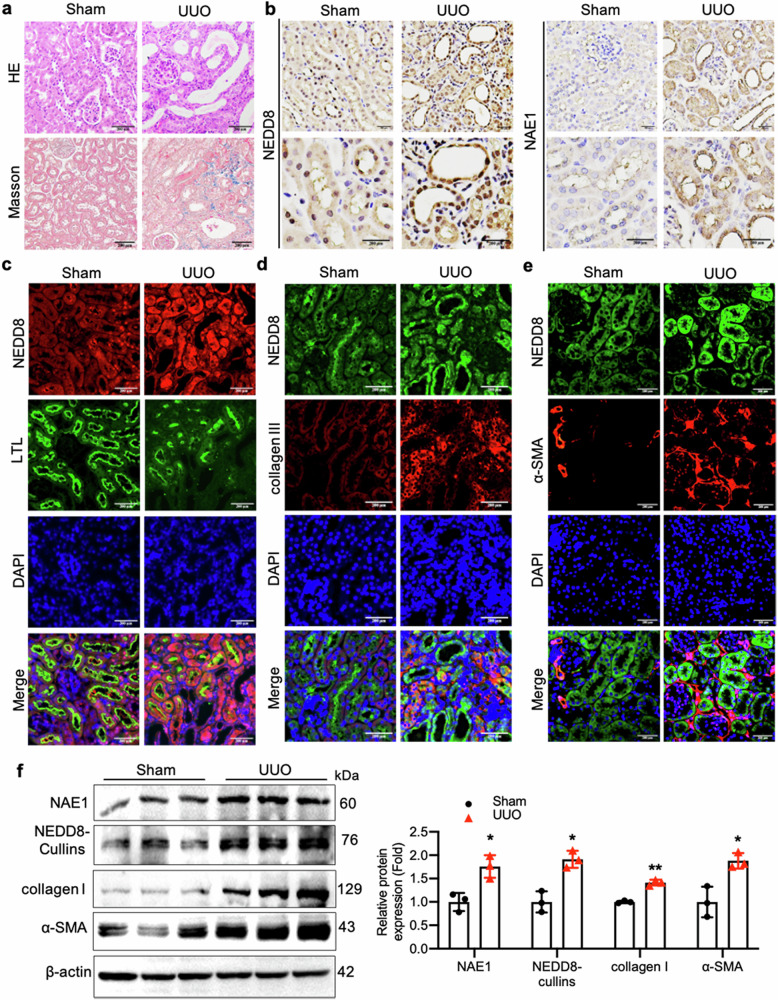
Hyper-neddylation is observed in mouse fibrotic kidneys subjected to unilateral ureteral ligation (UUO). **a** Representative HE and Masson staining images of kidney sections. *n* = 3 mice/group. Scale bar = 200 μm. **b** Immunohistochemistry of the kidney sections from mice subjected to UUO for 7 days. *n* = 3 mice/group. Scale bar = 200 μm. **c** Representative immunofluorescence images stained with NEDD8 (red), LTL (green), and DAPI (blue) of the cortical kidney sections from mice after UUO for 7 days. *n* = 3 mice/group. Scale bar = 200 μm. **d**, **e** Representative immunofluorescence images of the cortical kidney sections stained with NEDD8 (green), collagen III (red) or α-SMA (red), and DAPI (blue). *n* = 3 mice/group. Scale bar = 200 μm. **f** Western blot analysis of collagen I, α-SMA, NAE1, and NEDD8-cullins levels in the kidney tissues obtained from mice after UUO for 7 days. *n* = 3, mean ± SD, *P < 0.05, **P < 0.01.

To investigate the role of NAE1 and NEDD8 in kidney fibrosis, we measured their expression in another fibrotic mouse model induced by UUO. Seven days after UUO surgery, kidney injury was evident, as shown by H&E and Masson staining (Fig. 2a). Subsequently, we assessed NAE1 and NEDD8 expression in the renal tubules of UUO mice (Fig. 2b). Immunofluorescence analysis revealed that NEDD8 levels were significantly elevated in the renal tubules of UUO kidneys. These increased NEDD8 levels were primarily co-localized with the proximal tubular marker LTL, as well as collagen III and α-SMA, which are fibrosis markers, in the UUO-induced fibrotic kidneys (Fig. 2c–e). Quantitative data from Western blotting further supported the upregulation of NAE1 and NEDD8 expression in UUO kidneys (Fig. 2f). These findings suggest that the overactive neddylation pathway plays a significant role in the development of kidney fibrosis.

### Genetic knockdown or pharmacological inhibition of NAE1 mitigates the fibrotic phenotype in HK-2 cells

**Fig. 3:**
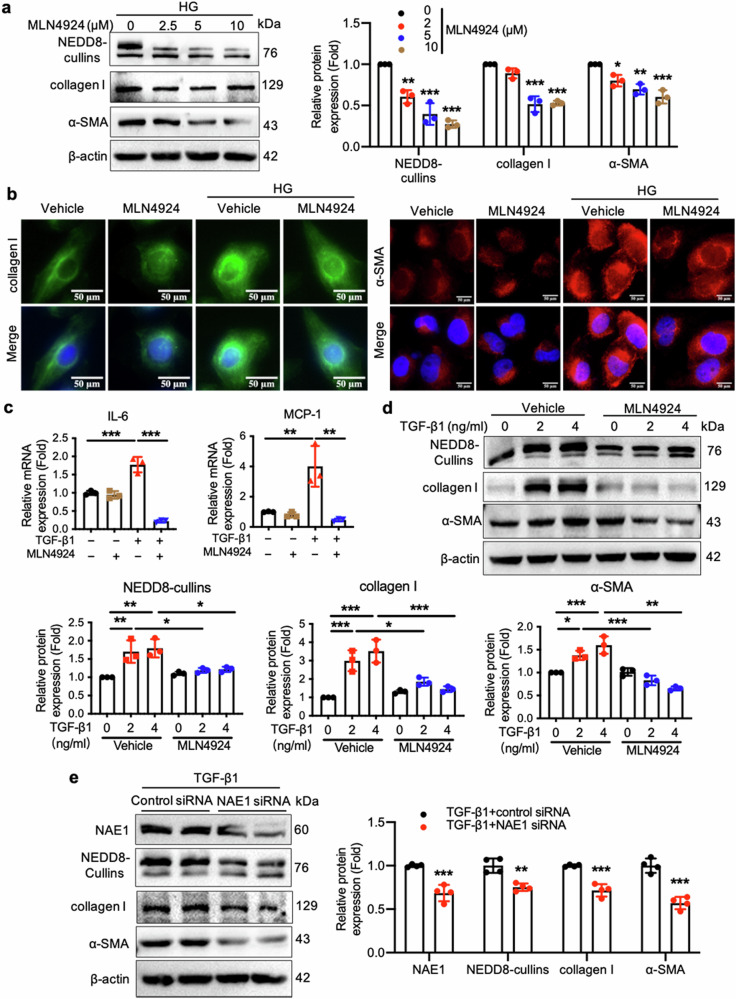
NAE1 silencing and MLN4924 attenuate profibrotic factors expression and inflammation in HG- or TGF-β1-induced HK-2 cells. **a** Immunoblotting assay for NEDD8-cullins, collagen I, and α-SMA of lysates from HG-induced HK-2 cells treated with vehicle or MLN4924 for the indicated different doses. *n* = 3, mean ± SD, *P < 0.05, **P < 0.01, ***P < 0.001. **b** Representative immunofluorescence images stained with collagen I (green), α-SMA (red), and DAPI (blue) in HG-induced HK-2 cells treated with vehicle or MLN4924 (10 μmol/L) for 24 h. Scale bar =  50 or 100 μm. **c** IL-6 and MCP-1 mRNA expression in TGF-β1-induced HK-2 cells treated with vehicle or MLN4924 (10 μmol/L) for 24 h. *n* = 3, mean ± SD, **P < 0.01, ***P < 0.001. **d** Representative immunoblot and quantification of NEDD8-conjugated cullins, collagen I, and α-SMA in TGF-β1-induced HK-2 cells treated with vehicle or MLN4924 (10 μmol/L) for 24 h. *n* = 3, mean ± SD, *P < 0.05, **P < 0.01, ***P < 0.001. **e** Representative immunoblot of NAE1, NEDD8-conjugated cullins, collagen I, and α-SMA in TGF-β1-induced HK-2 cells transfected with control siRNA or NAE1 specific siRNA for 24 h. *n* = 4, mean ± SD, **P < 0.01, ***P < 0.001.

Our findings suggest that renal tubules are a primary site for neddylation activation in response to UUO. To further explore the functional roles of NAE1, we used HK-2 cells as an in vitro model. Pharmacological inhibition of NAE1 in HG-stimulated HK-2 cells was achieved using MLN4924. As depicted in Fig. 3a, b, MLN4924 effectively suppressed HG-induced expression of collagen I and α-SMA in HK-2 cells. Additionally, MLN4924 treatment reduced the mRNA expression of inflammatory cytokines IL-6 and monocyte chemoattractant protein-1 (MCP-1) in HG-treated HK-2 cells (Fig. 3c). Transforming growth factor-β1 (TGF-β1), a key contributor to CKD progression, was also used to induce injury in HK-2 cells. MLN4924 successfully inhibited TGF-β1-induced expression of collagen I and α-SMA in the stimulated HK-2 cells (Fig. 3d). Furthermore, NAE1 knockdown using siRNA mirrored the effects of MLN4924 in vitro, restoring the TGF-β1-induced upregulation of profibrotic markers collagen I and α-SMA in HK-2 cells (Fig. 3e). These results highlight the significance of NAE1 in mediating the profibrotic effects of TGF-β1.

### AAV9-mediated silencing of NAE1 protects against kidney injury and renal tubulointerstitial fibrosis in a diabetic mouse model

**Fig. 4:**
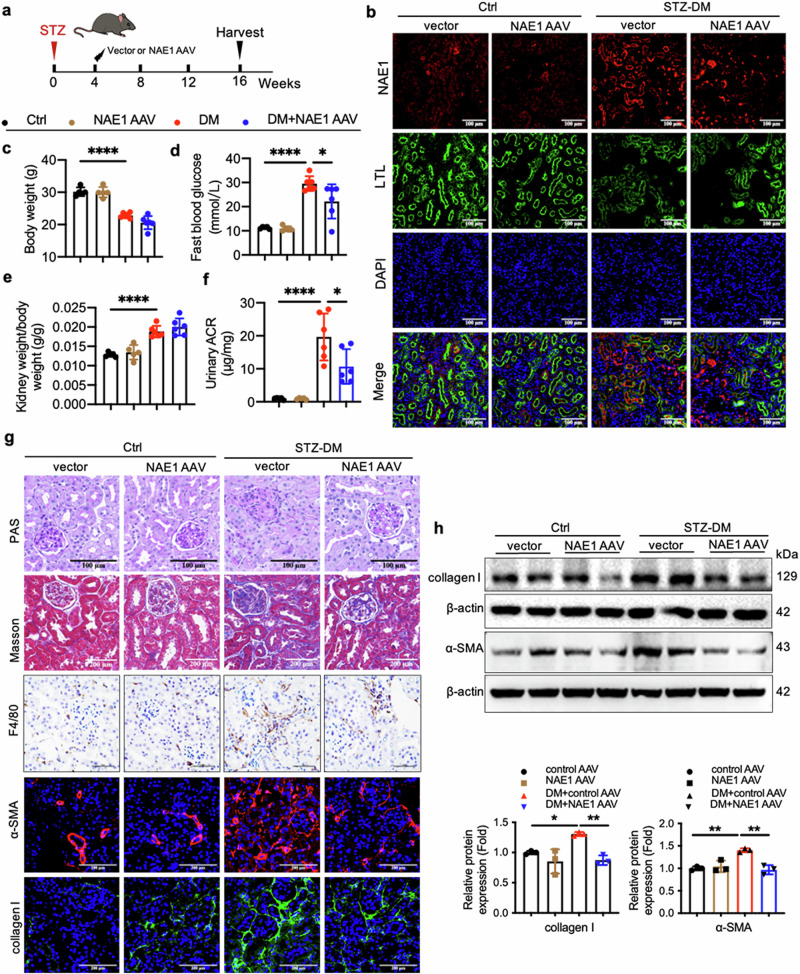
AAV9-mediated silencing of NAE1 protects against kidney injury and renal tubulointerstitial fibrosis in a diabetic mice model. Two weeks after the successful construction of a diabetes mouse model, the mice were randomly assigned to four groups and administered tail vein injection of AAV9 encoding NAE1 or scramble. The four groups were as follows: (Ctrl) (*n* = 5), NAE1 AAV (*n* = 5), diabetes (DM) (*n* = 6), and NAE1 AAV with diabetes (NAE1 AAV + DM) (*n* = 6). The mice were sacrificed at week 16. mean ± SD, *P < 0.05, **P < 0.01, ****P < 0.0001. **a** The timeline diagram depicts the animal experimental procedure. **b** Representative immunofluorescence images stained with NAE1 (red), LTL (green), and DAPI (blue) of the cortical kidney sections. Scale bar = 100 μm. Body weight (**c**) and fasting blood glucose level (**d**) of the mice in the four groups. **e** Kidney weight to body weight ratio of the mice in the four groups. **f** Detection of the urinary albumin-to-creatinine ratio (ACR). **g** PAS and Masson staining, immunohistochemistry of F4/80, and the co-immunohistochemistry of α-SMA (red) and collagen I (green) of kidneys of mice. Scale bar = 100 or 200 μm. **h** Western Blot and quantitative data analyses of collagen I and α-SMA.

Given the hyperactivation of neddylation in RTECs and its potential role in DN, we employed an AAV9-packaged NAE1 knockdown plasmid to assess its therapeutic potential in diabetic mice. As depicted in Fig. 4a, AAV9 efficiently delivered the NAE1 knockdown construct, resulting in reduced NAE1 expression in RTECs, as confirmed through immunofluorescence (Fig. 4b). In diabetic mice, STZ administration led to significant weight loss (Fig. 4c) and hyperglycemia (Fig. 4d). Although AAV9-NAE1 treatment did not alter the kidney weight-to-body weight (KW/BW) ratio (Fig. 4e), it significantly reduced urinary albumin levels, indicating a protective effect against albuminuria (Fig. 4f). Histopathological analysis revealed that diabetic mice exhibited glomerular hypertrophy, mesangial matrix expansion, and tubulointerstitial fibrosis, as shown by PAS and Masson staining (Fig. 4g). NAE1 knockdown alleviated these pathological changes, along with a reduction in urinary albuminuria. Western blot analysis further confirmed a reduction in collagen I and α-SMA protein levels (Fig. 4h). These results demonstrate that NAE1 silencing protects against renal injury and fibrosis in diabetic kidneys, highlighting its potential as a therapeutic target for DN.

### Interaction of NEDD8 with RhoA impedes the degradation of the RhoA protein

**Fig. 5:**
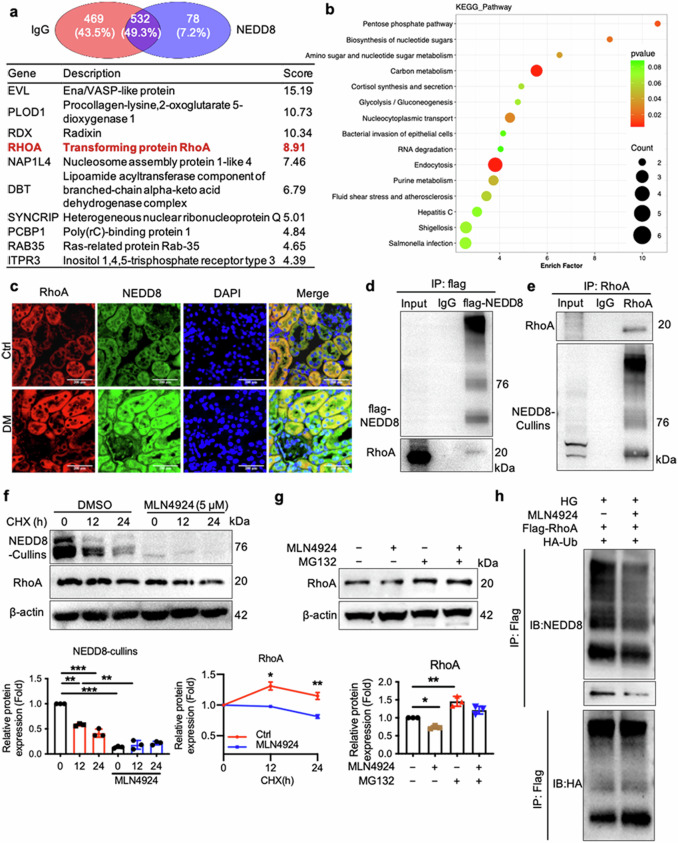
NEDD8 directly interacts with RhoA and regulates the protein stability of RhoA. **a** The Venn diagram displays the numbers of binding proteins immunoprecipitated with NEDD8 by IP/MS analysis. The top 10 proteins are presented. **b** The correlational analysis identified the top 14 enriched KEGG pathways that were potentially involved in NEDD8-related substrate proteins. **c** Representative images of the STZ-induced cortical kidney sections stained with NEDD8 (green), RhoA (red), and DAPI (blue). Scale bar = 200 μm. **d** Co-IP assays of lysates from HEK293T cells co-expressing flag-NEDD8 were performed to validate the NEDD8/RhoA complex formation at the exogenous level. **e** IP assays of lysates from HK-2 cells were performed to confirm the NEDD8/RhoA complex formation at the endogenous level. **f** CHX chase assays and Western blot analysis of RhoA were performed to monitor the half-life of the RhoA protein in MLN4924-treated HK-2 cells and vehicle control cells. Each group of cells were treated with CHX (50 μg/mL) for the indicated time period. *n* = 3, mean ± SD, *P < 0.05, **P < 0.01, ***P < 0.001. **g** MLN4924-treated HK-2 cells for 18 h and then treated with MG132 (2 μmol/L) for an additional 6 h, followed by subjecting to Western Blot for the RhoA expression. *n* = 3, mean ± SD, *P < 0.05, **P < 0.01. **h** IP assays of lysates from HK-2 cells were performed to confirm the changes of neddylation and ubiquitination of RhoA. HK-2 cells were collected after transfection with Flag-RhoA and HA-ubiquitin plasmids, with or without MLN4924 treatment.

NEDD8, a ubiquitin-like protein, covalently binds to substrate proteins, suggesting its involvement in renal fibrosis through protein stabilization. To identify NEDD8-cullin interacting proteins, we performed Co-IP combined with mass spectrometry (MS) analysis. This revealed a strong interaction between NEDD8-cullins and RhoA in HK-2 cells (Fig. 5a). KEGG pathway analysis of differentially expressed proteins from MS data identified top 14 enriched KEGG pathways that may be involved in NEDD8-related substrate proteins, including the endocytosis signaling pathway, which is potentially relevant to NEDD8-mediated effects (Fig. 5b). RhoA, a predominant Rho-GTPase in renal proximal tubules, regulates cytoskeleton rearrangement and migration [24] and is implicated in tubulointerstitial fibrosis in DN models [25–27]. We found that NEDD8 and RhoA co-localization in RTECs was significantly upregulated in diabetic mice (Fig. 5c). Co-IP experiments further confirmed the direct interaction between NEDD8 and RhoA, as Flag-tagged NEDD8 bound directly to RhoA in HEK293T cells (Fig. 5d). Similarly, endogenous NEDD8 was immunoprecipitated by anti-NEDD8 antibody, and endogenous RhoA protein was detected in HK-2 cells. These two endogenous forms were detected as interacting proteins in HK-2 cells (Fig. 5e). Overall, these findings indicate that RhoA is a substrate of NEDD8. To determine whether neddylation stabilizes RhoA protein, we conducted cycloheximide (CHX) assays, which demonstrated that MLN4924-induced neddylation inhibition shortened RhoA’s half-life and significantly accelerated its degradation (Fig. 5f). Furthermore, MLN4924-mediated RhoA degradation was partially blocked by the proteasome inhibitor MG132, indicating a role for the ubiquitin-proteasome pathway (Fig. 5g). Overexpression of Flag-RhoA and HA-ubiquitin plasmids in HK-2 cells confirmed that MLN4924 reduced RhoA neddylation while increasing its ubiquitination (Fig. 5h). Taken together, these findings reveal that the neddylation pathway stabilizes RhoA by inhibiting its ubiquitin-proteasome-mediated degradation.

### The stability of RhoA triggered by the neddylation promotes renal fibrosis

**Fig. 6:**
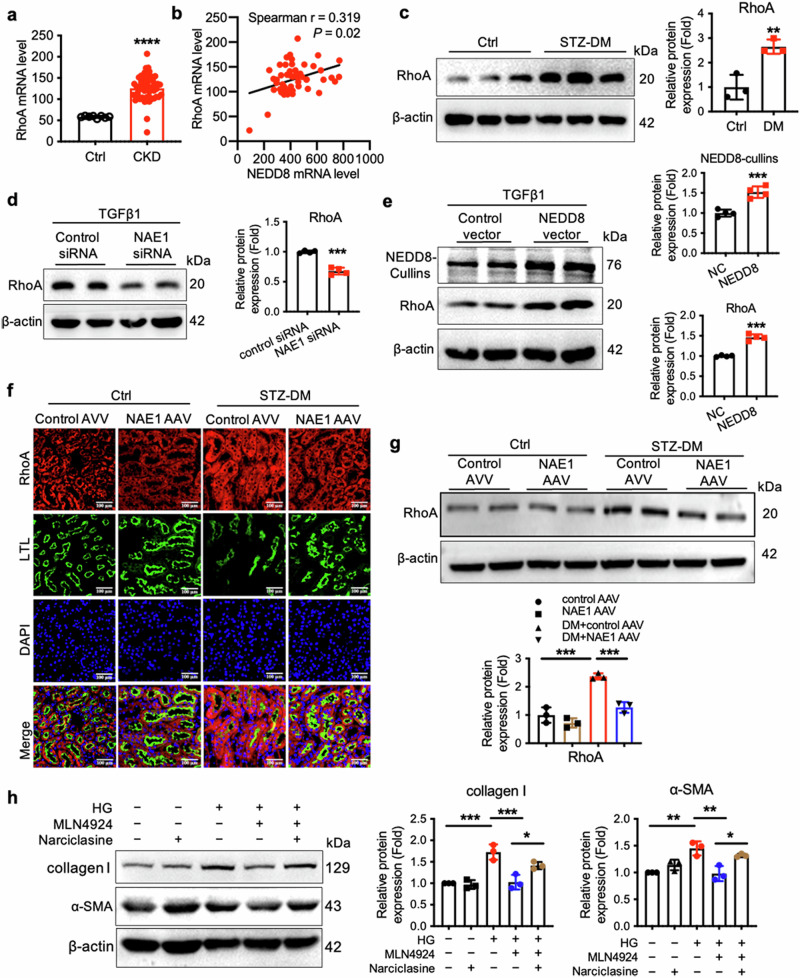
Neddylation-mediated fibrosis progression depends on the RhoA level. **a** The mRNA levels of RhoA in the kidney specimens of CKD (*n* = 53 samples) and the controls (*n* = 8 samples) in the GSE66494 dataset (Probe ID: A_23_P69491). mean ± SD, ****P < 0.0001. **b** The correlation between RhoA and NEDD8 mRNA expression in human clinical kidney specimens of CKD (*n* = 53 samples). **c** Western blot of RhoA in the cortical kidney tissues of STZ-induced diabetic mice. *n* = 3, mean ± SD, **P < 0.01. **d** Representative immunoblot of RhoA in TGF-β1-induced HK-2 cells transfected with control siRNA or NAE1-specific siRNA for 24 h. *n* = 4, mean ± SD, ***P < 0.001. **e** Western blot analysis of NEDD8-cullins and RhoA in TGF-β1-induced HK-2 cells transfected with control vector or NEDD8 overexpression vector for 24 h. *n* = 4, mean ± SD, ***P < 0.001. **f** Coimmunostaining with RhoA (red) and LTL (green) on the kidneys of different groups of mice. Scale bar = 100 μm. **g** Immunoblotting assay for RhoA of lysates from the kidneys of different groups of mice. *n* = 3, mean ± SD, ***P < 0.001. **h** MLN4924 inhibited the expression of pro-fibrotic factors collagen I and α-SMA in TGF-β1-induced HK-2 cells, which was reversed by RhoA activator narciclasine. *n* = 3, mean ± SD, *P < 0.05, **P < 0.01, ***P < 0.001.

Given the marked influence of NEDD8 on RhoA protein stability in renal fibrosis, we investigated its role in the regulation of RhoA protein expression during profibrotic pathogenesis. Analysis of the GSE66494 dataset revealed elevated RhoA mRNA levels across various CKD types, including DN, compared with healthy controls (*n* = 53 CKD, *n* = 8 healthy controls) (Fig. 6a). Additionally, a positive correlation was observed between RhoA mRNA expression and NEDD8 mRNA expression in CKD patients (Fig. 6b). In STZ-induced diabetic mice, we observed a marked increase in RhoA protein levels in fibrotic kidneys (Fig. 6c). Both in vivo *and* in vitro, NAE1 knockdown reduced RhoA protein levels (Fig. 6d). In HK-2 cells transfected with a Flag-tagged NEDD8 plasmid, the protein levels of exogenous RhoA increased significantly and proportionally with the expression of exogenous NEDD8 (Fig. 6e–g), indicating that NEDD8 stabilizes RhoA. To further investigate the role of RhoA in fibrosis, we used narciclasine, a specific activator of RhoA, to enhance fibrotic protein expression (Fig. 6h). Consequently, the inhibition of NAE1 partially suppressed TGF-β1-induced profibrotic phenotypes in HK-2 cells by promoting RhoA protein degradation.

### Neddylation activates RhoA and the ERK1/2 signaling pathway

**Fig. 7:**
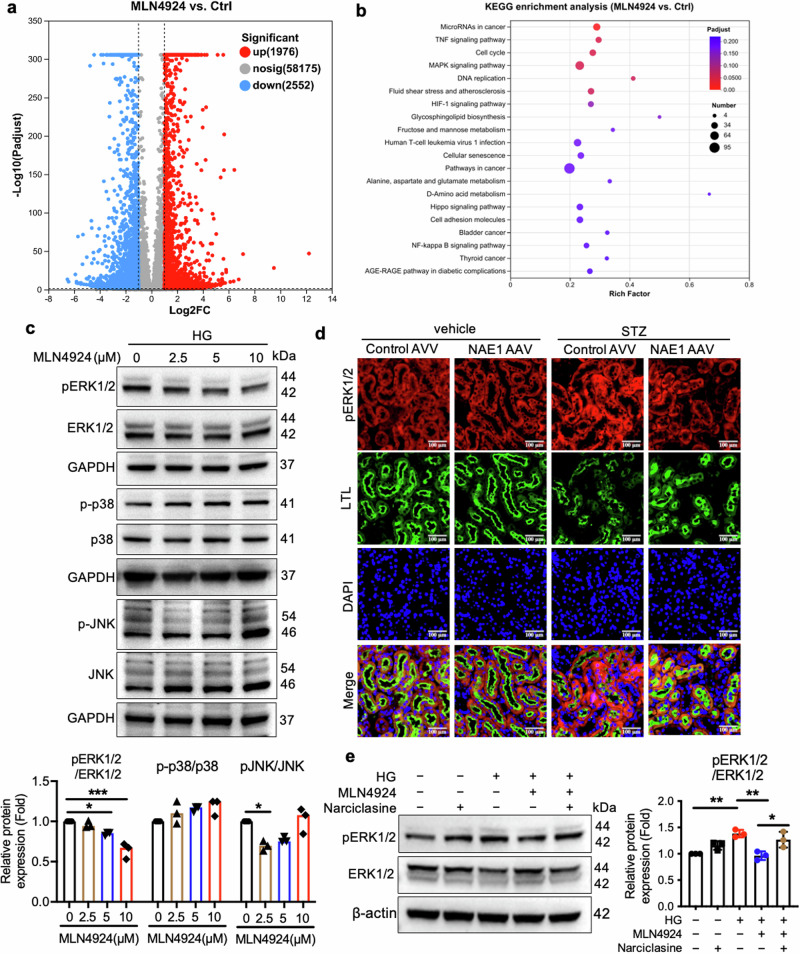
ERK1/2 pathway activation is involved in RhoA-upregulation-mediated fibrosis progression. **a** Volcano map depicting differential genes obtained from RNA sequencing. **b** Heat map illustrating the differential gene expression profiles of HK-2 cells with or without MLN4924 treatment (*n* = 6 for each group). **c** Immunoblotting assay for pERK1/2, ERK1/2, pJNK, JNK, p-p38, and p38 of lysates from HK-2 cells treated with vehicle or MLN4924 for the indicated different doses. *n* = 3, mean ± SD, *P < 0.05, ***P < 0.001. **d** Coimmunostaining with pERK1/2 (red) and LTL (green) on the kidneys from different groups of mice. Scale bar = 100 μm. **e** Immunoblotting assay for pERK1/2, ERK1/2 of lysates from HK-2 cells treated with vehicle, MLN4924, or narciclasine. *n* = 3, mean ± SD, *P < 0.05, **P < 0.01.

To elucidate the signaling mechanisms underlying neddylation-induced fibrosis in RTECs, we conducted RNA-seq analysis on HK-2 cells treated with vehicle or MLN4924 (Fig. 7a). KEGG analysis revealed enrichment in pathways related to cancer, mitogen-activated protein kinases (MAPKs), TNF signaling, and the cell cycle (Fig. 7b). Because MAPKs were prominently enriched, we analyzed ERK1/2, JNK, and p38 phosphorylation in MLN4924-treated HK-2 cells. MLN4924 selectively inhibited ERK1/2 phosphorylation without affecting JNK or p38 activation (Fig. 7c). In diabetic mouse kidneys, phosphorylated ERK1/2 levels were elevated, as evidenced by increased immunofluorescence intensity, while NAE1 knockdown reduced this signal (Fig. 7d). Narciclasine, a specific RhoA activator, increased ERK1/2 phosphorylation, an effect suppressed by MLN4924 (Fig. 7e). These findings suggest that neddylation enhances RhoA activity, which in turn activates the ERK1/2 pathway.

### The NAE1 inhibitor MLN4924 exerts renoprotective effects in a UUO mouse model

**Fig. 8:**
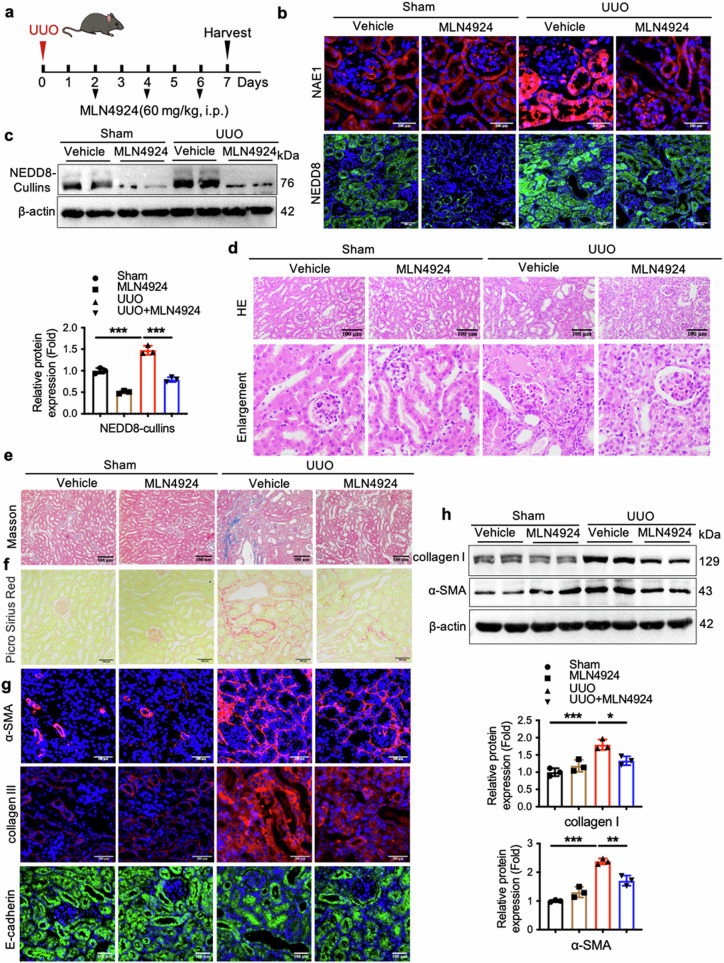
NAE1 inhibitor MLN4924 reduces UUO-induced mouse renal interstitial fibrosis. The experimental mice were assigned to four groups: mice with sham operation (Sham, *n* = 6), mice with sham operation and MLN4924 injection (MLN4924, *n* = 6), mice subjected to UUO operation (UUO, *n* = 6), and mice with UUO operation and MLN4924 injection (UUO + MLN4924, *n* = 6). The mice were sacrificed 7 days after the UUO operation. **a** A schematic diagram of a mouse model with UUO operation. **b** Representative immunofluorescence images stained with NAE1 (red), NEDD8 (green), and DAPI (blue) of the cortical kidney sections from mice. Scale bar= 200 or 100 μm. **c** The expression of NEDD8-cullins in the kidney tissues of mice, as determined by Western blot. *n* = 3, mean ± SD, ***P < 0.001. Paraffin-embedded kidney sections were stained with HE (**d**), Masson (**e**), and Sirius red staining (**f**). Scale bar = 100 or 200 μm. **g** Representative immunofluorescence images stained with α-SMA (red), collagen III (red), E-cadherin (green), and DAPI (blue) of the cortical kidney sections from mice. Scale bar = 200 or 100 μm. **h** Kidney collagen I and α-SMA protein expression was determined by Western blot. *n* = 3, mean ± SD, *P < 0.05, **P < 0.01, ***P < 0.001.

We evaluated the therapeutic potential of MLN4924, a neddylation inhibitor, in mitigating renal fibrosis using a UUO model. Following surgery, mice received intraperitoneal injections of MLN4924 (60 mg/kg body weight) every other day for three doses (Fig. 8a). MLN4924 treatment effectively reduced NAE1 expression and NEDD8-conjugated protein levels in renal tubular cells of UUO kidneys (Fig. 8b, c). Histological analyses revealed significant improvements in renal damage with MLN4924, as evidenced by reduced tubular injury and inflammation on H&E staining (Fig. 8d). Fibrosis markers were significantly decreased in the MLN4924-treated mice compared with the control mice, as evidenced by Masson and Sirius red staining (Fig. 8e, f). At the molecular level, MLN4924 restored E-cadherin protein levels and reduced the expression of profibrotic markers, such as collagen I and α-SMA, as confirmed by Western blot analysis (Fig. 8g, h). These findings collectively demonstrate that MLN4924, an inhibitor of the NEDD8-activating enzyme, effectively attenuates renal tubulointerstitial fibrosis in UUO mice by inhibiting the neddylation pathway.

## Discussion

This study demonstrates that neddylation is upregulated in fibrotic kidneys and is particularly active in RTECs, driving DN progression. Through analysis of previously published microarray datasets, we observed NAE1 and NEDD8 overexpression in CKD. Examination of renal biopsy samples from DN patients, supported by renal histopathological analysis, confirmed that hyperactivation of the neddylation pathway contributes significantly to CKD progression. Importantly, we found that either NAE1 genetic knockdown or pharmacological inhibition with the neddylation inhibitor MLN4924 effectively attenuated renal tubular injury, demonstrating therapeutic potential. MLN4924, a neddylation inhibitor currently in phase I/II clinical trials for cancer treatment, may serve as a promising therapeutic agent for renal interstitial fibrosis. Mechanistically, cellular injury activates the substrate of the neddylation pathway, allowing NEDD8 to directly interact with and stabilize RhoA by reducing its ubiquitination-mediated degradation. Our findings demonstrate that this stabilization promotes renal interstitial fibrosis via activation of the ERK1/2 signaling pathway.

To further explore the role of neddylation in DN, we used AAV9-mediated NAE1 knockdown in diabetic mice. Mice with silenced NAE1 exhibited significantly reduced renal tubular damage and interstitial fibrosis. These observations align with findings in fibrotic kidneys of DN patients, which exhibited elevated NAE1 expression. Moreover, NAE1-deficient diabetic mice were protected against renal tubular lesions and renal interstitial fibrosis, underscoring the pathway’s critical pro-fibrotic role. MLN4924 was specifically designed as a first-in-class pharmacological inhibitor of NAE1 and has been investigated in various phase I–III clinical trials for leukemia, lymphoma, and advanced solid tumors [28]. In our study, immunoblotting assays revealed that MLN4924 significantly suppresses protein neddylation, mirroring the renoprotective effects of NAE1 gene knockdown both in vitro and in vivo. By inhibiting the RhoA/ERK1/2 pathway, MLN4924 reduced collagen accumulation and inflammatory cell infiltration, alleviating renal interstitial fibrosis. These results strongly suggest that NAE1 is a viable therapeutic target for renal fibrosis, and MLN4924 holds promise as a novel antifibrotic agent.

To elucidate the mechanisms by which neddylation promotes fibrosis, we identified neddylation-modified substrates through Co-IP/MS analysis in HG-stimulated HK-2 cells. Our findings confirmed the direct interaction between NEDD8 and RhoA, validated by both endogenous and exogenous IP assays in vitro. RhoA, a small GTPase involved in cellular motility, migration, and cytoskeletal organization [28, 29], plays a pivotal regulatory role in fibrosis due to its wide expression in various tissues, including the kidneys [30]. Studies have focused on the ubiquitination-mediated degradation of RhoA, which precisely controls its protein levels and influences its downstream signaling pathway [31]. RhoA protein levels are tightly regulated through ubiquitination by E3 ligases such as the HECT type E3 smad ubiquitination regulatory factor 1 (Smurf1) [32] and CRLs [26], leading to its degradation. However, the role of neddylation in RhoA stabilization has remained unexplored. Our study demonstrates that neddylation stabilizes RhoA in renal tubular cells under diabetic conditions, preventing its degradation and amplifying profibrotic signaling via the ERK1/2 pathway. Attenuating neddylation reduced renal fibrosis through RhoA-ERK1/2 dependent mechanisms.

To validate the clinical implications, we compared RhoA expression in the kidneys of CKD patients to that of healthy controls. Our findings unveiled that patients with CKD exhibit higher RhoA expression in the kidneys. Furthermore, an analysis of a publicly available dataset revealed a positive correlation between the mRNA levels of NEDD8 and RhoA, underscoring the functional link between neddylation and RhoA expression. These observations highlight the potential of targeting the neddylation pathway of RhoA as a novel therapeutic strategy for combating renal fibrosis. Interestingly, we observed that knockdown of NAE1, a key regulatory subunit of the NEDD8 E1 enzyme, increased RhoA protein levels and enhanced ERK1/2 phosphorylation. These results suggest a dual role for NAE1: not only does it modulate RhoA stability but also influences the activation of the ERK1/2 pathway, providing further mechanistic insights into the interplay between neddylation and profibrotic signaling pathways.

PTMs involve covalent alterations to proteins or peptides, such as proteolytic cleavage or the addition of functional groups moieties to specific amino acids, and they play critical roles in regulating protein expression and function [33, 34]. The interplay of multiple PTMs, including neddylation, sumoylation, acetylation, and methylation, adds complexity to protein regulation in CKD development [34, 35]. Notably, neddylation, in conjunction with other established PTMs such as sumoylation, acetylation, and methylation [36], plays crucial roles in DN pathogenesis. In conjunction with prenylation [37] and ubiquitination [38], our study posits that neddylation represents a novel form of PTM for RhoA involved in DN progression. By uncovering the regulatory mechanisms of RhoA stability and function through neddylation, we provide a new conceptual framework for comprehending its role in DN development and progression.

This study demonstrates that silencing NAE1 in RTECs leads to the loss of neddylation, promoting RhoA degradation and subsequent inactivation of the ERK1/2 pathway. This mechanistic shift ultimately ameliorates renal fibrosis in both diabetic and UUO mouse models. These findings reveal an unrecognized role for neddylation in renal fibrosis, positioning it as a crucial driver of DN pathogenesis. Moreover, the identification of neddylation as a novel PTM pathway of RhoA opens the door to precision therapy. Targeting RhoA neddylation could represent a transformative therapeutic approach to managing renal fibrosis.

### Limitations

A notable observation was that NAE1 knockdown reduced fasting blood glucose levels in diabetic mice, suggesting a potential role for NAE1 in glucose metabolism. This observation indicates that its renal protective effects may be partially mediated by metabolic regulation. Future studies will further investigate the impact of neddylation on glucose and lipid metabolism to clarify the relationship. Additionally, tubule-specific deletion of NAE1 in mice or the use of tubule-specific AAV9-mediated gene silencing would provide deeper insights into the precise role of NAE1 in tubular epithelial cells.

## Supplementary information

Supplementary Table 1

Original WB gels

## Data Availability

All data supporting the findings of this study are available within the manuscript and its supplementary files. The publicly available data on human renal transcriptomics used in this study are available in the Gene Expression Omnibus (GEO) database (accession code GSE66494).
