# Supplementary material for: Neddylation of RhoA impairs its protein degradation and promotes renal interstitial fibrosis progression in diabetic nephropathy
Source: Acta Pharmacol Sin. 2025 Feb 3;46(6):1692–705. doi: 10.1038/s41401-024-01460-z (PMC12098688; doi:10.1038/s41401-024-01460-z)
Supplement: Supplementary file 1 — Supplementary Table 1 [file 41401_2024_1460_MOESM1_ESM.docx]

**Construction of mouse *Nae1* shRNA adeno-associated virus vector**

shRNA1: GCGGAGAAGATGCTGGAAACATTCAAGAGATGTTTCCAGCATCTTCTCCGCTTTTTT

shRNA2: GGTTGCAACTCAGCTTCTTGATTCAAGAGATCAAGAAGCTGAGTTGCAACCTTTTTT

shRNA3: GCACAGTGGTATAATGAAACATTCAAGAGATGTTTCATTATACCACTGTGCTTTTTT

shRNA4: GCATTTCTTCGAGTGGTAAGATTCAAGAGATCTTACCACTCGAAGAAATGCTTTTTT


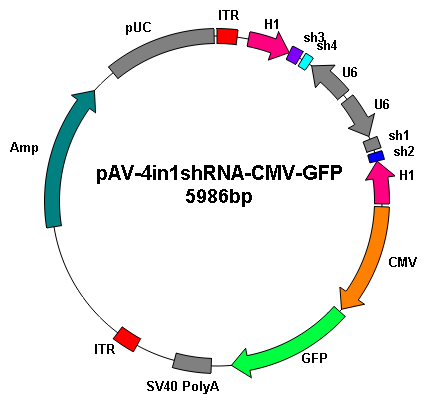


The siRNA sequences are listed below:

NAE1 siRNA-1: 5’-GAGGCACAAUUCCUGAUAUTT-3’;

NAE1 siRNA-2: 5’-AUAUCAGGAAUUGUGCCUCTT-3’.
