# Supplementary material for: Neddylation of RhoA impairs its protein degradation and promotes renal interstitial fibrosis progression in diabetic nephropathy
Source: Acta Pharmacol Sin. 2025 Feb 3;46(6):1692–705. doi: 10.1038/s41401-024-01460-z (PMC12098688; doi:10.1038/s41401-024-01460-z)

Figure 1A

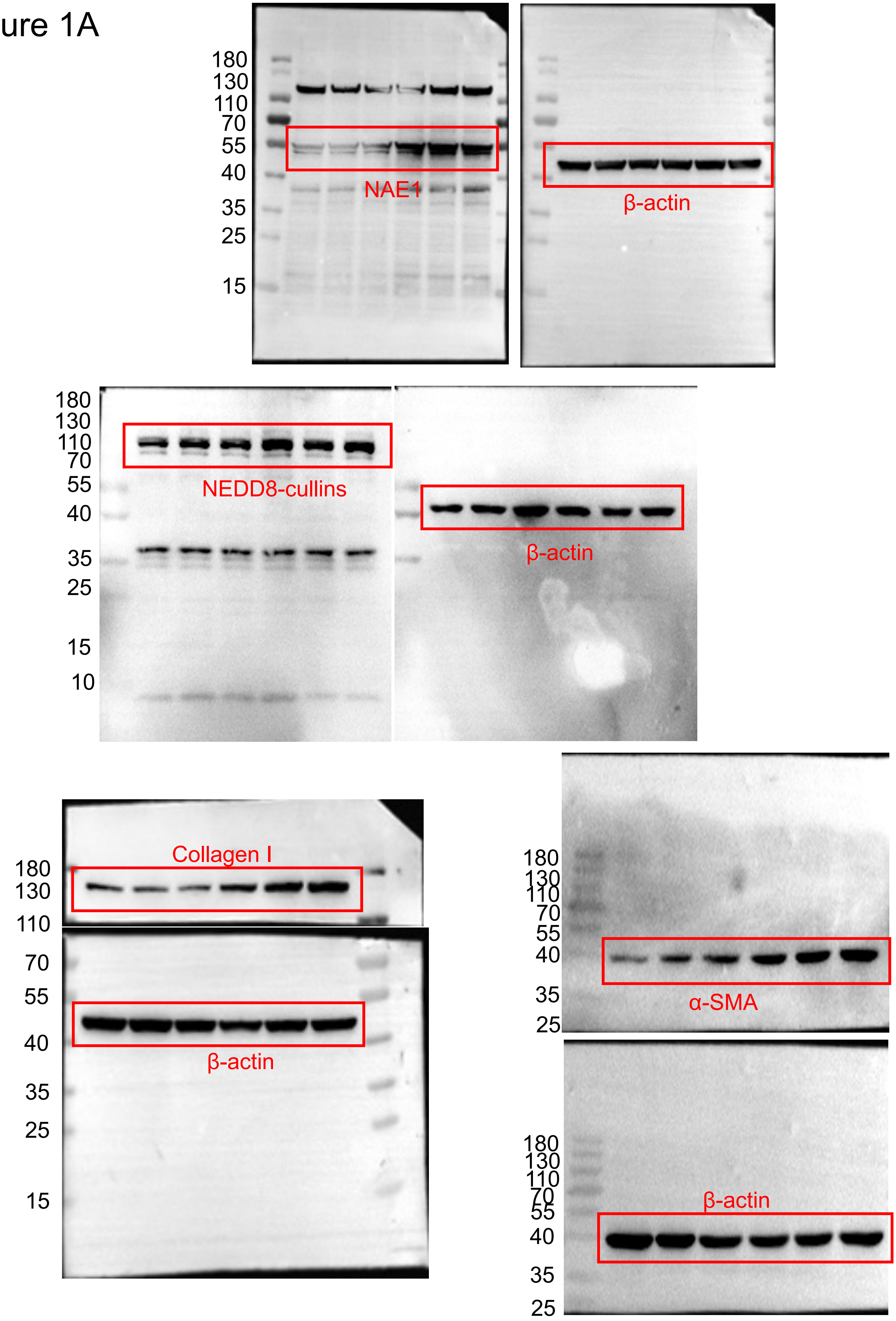

180
130
110
70
55
40
35
25
15
NAE1
β-actin
180
130
110
70
55
40
35
25
15
10
NEDD8-cullins
β-actin
Collagen I
180
130
110
70
55
40
35
25
15
β-actin
180
130
110
70
55
40
35
25
α-SMA
180
130
110
70
55
40
35
25
β-actin

Figure 2F

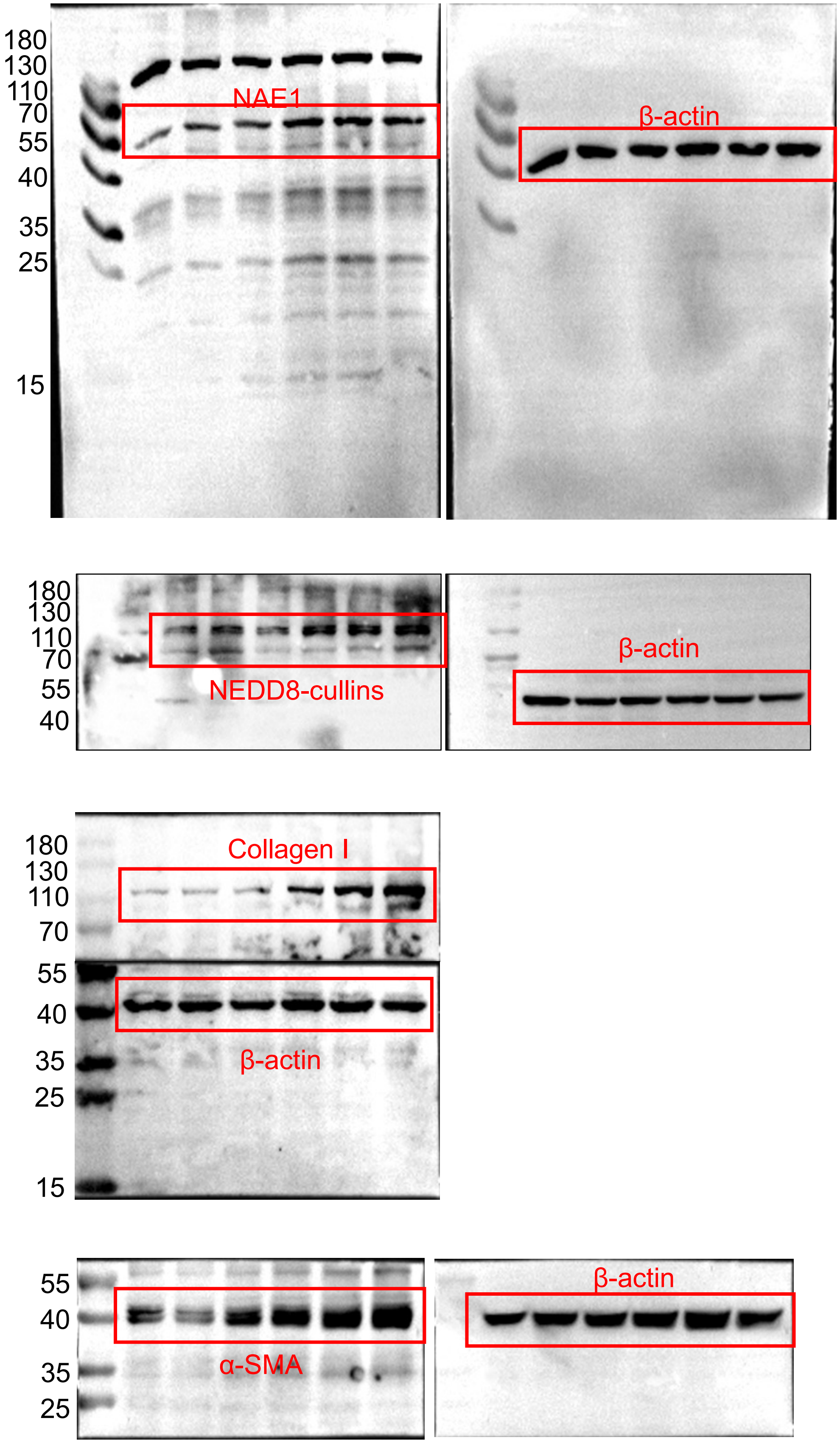
180
130
110
70
55
40
35
25
15
NAE1
β-actin
NEDD8-cullins
Collagen I
α-SMA

Figure 3A

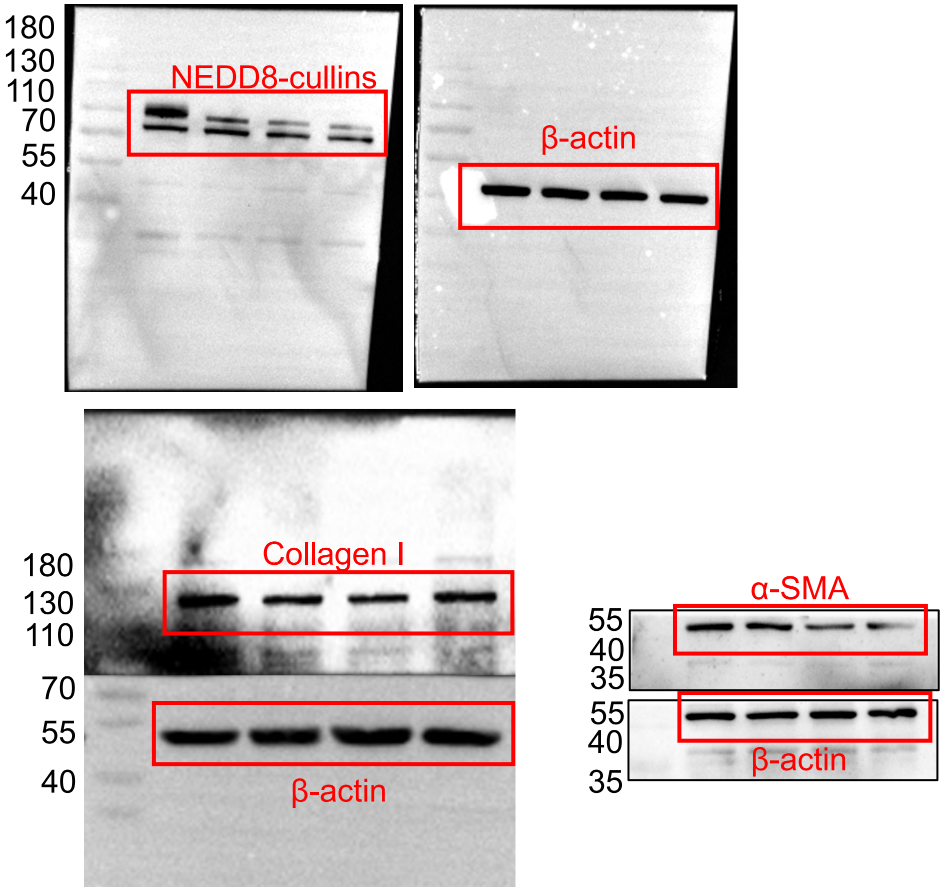

Figure 3D

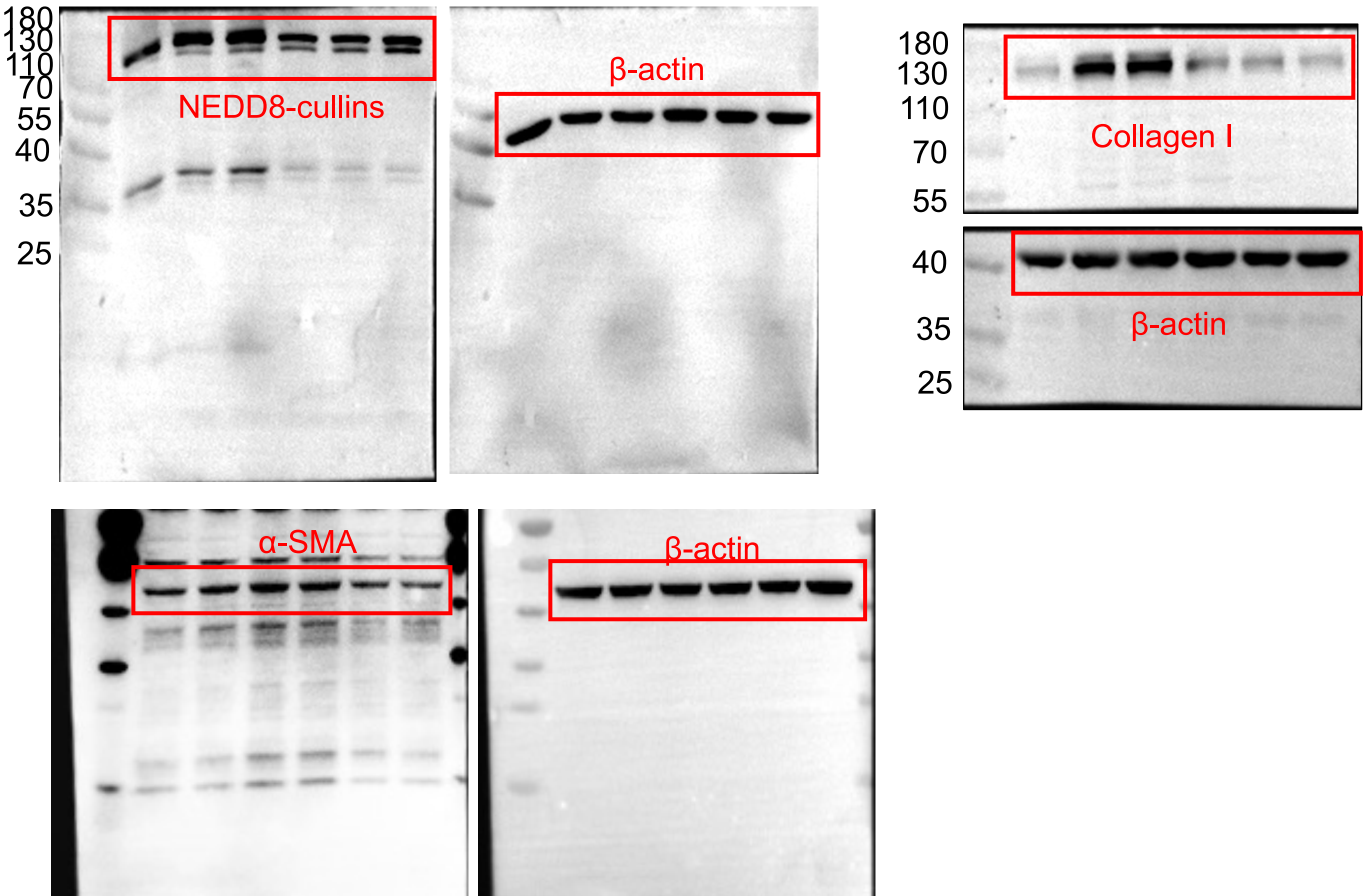

Figure 3E

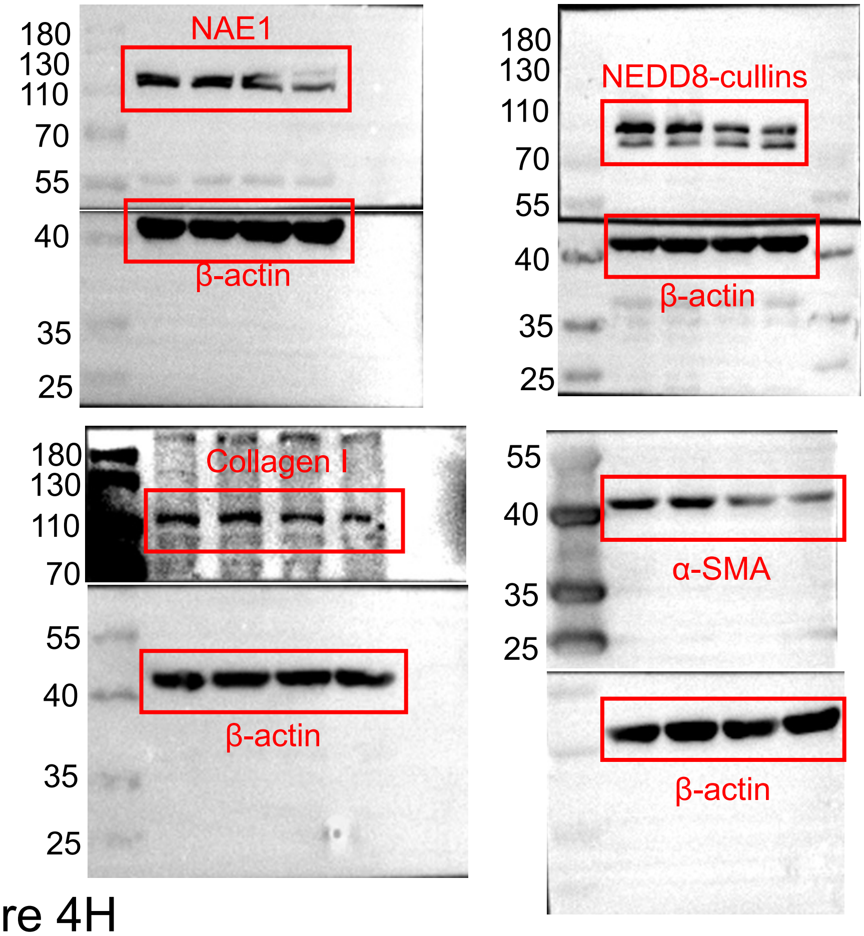

Figure 4H

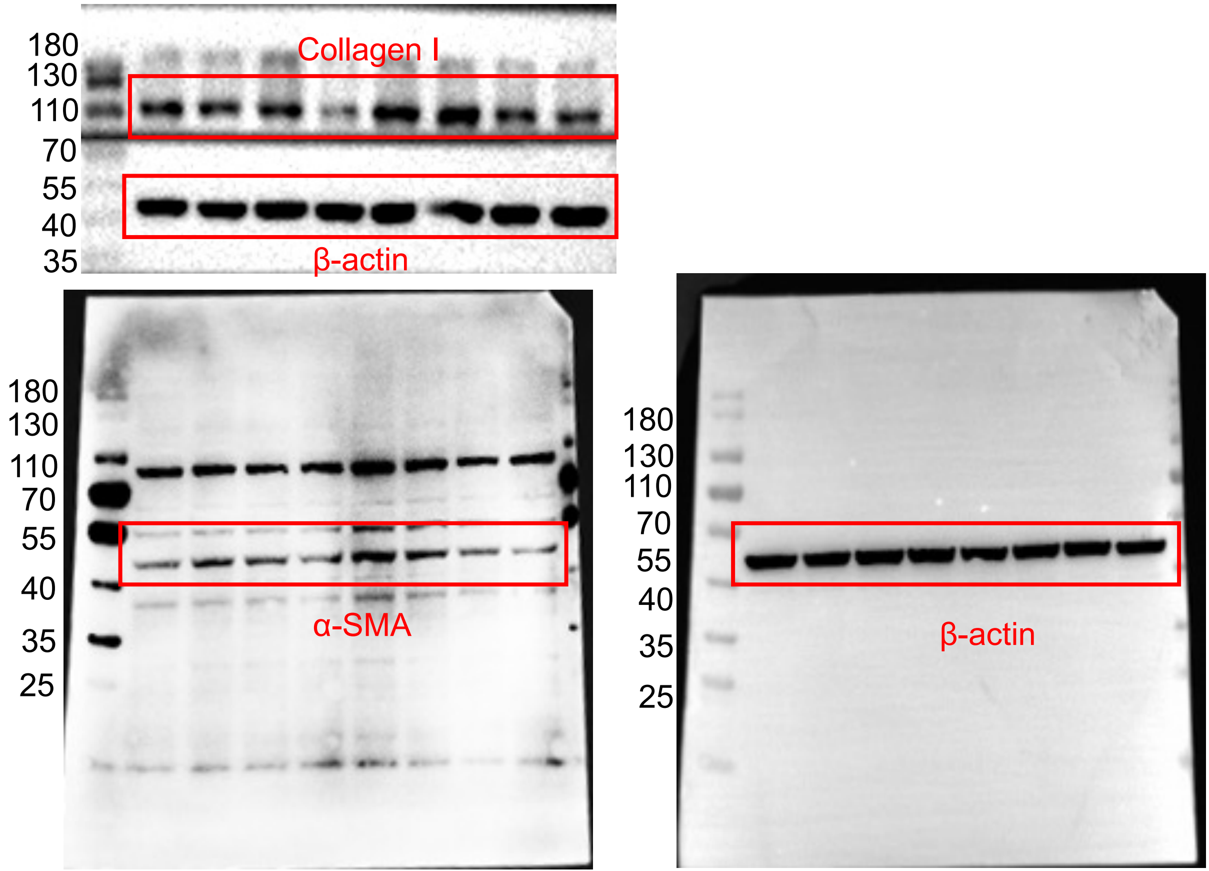

Figure 5D

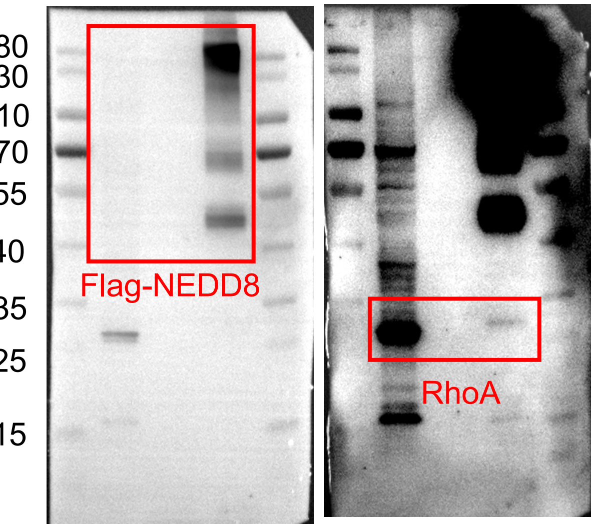

Figure 5E

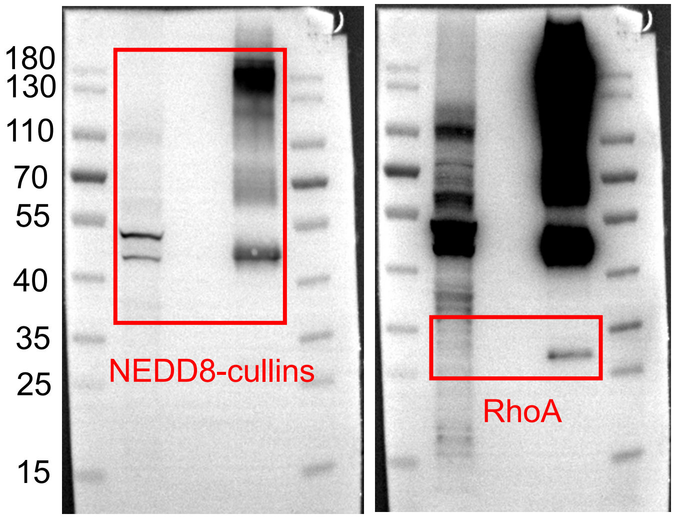

Figure 5F

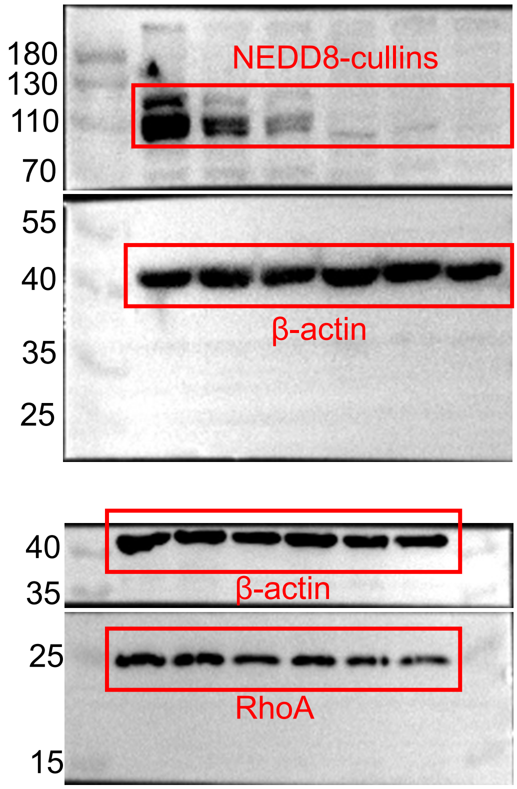

Figure 5G

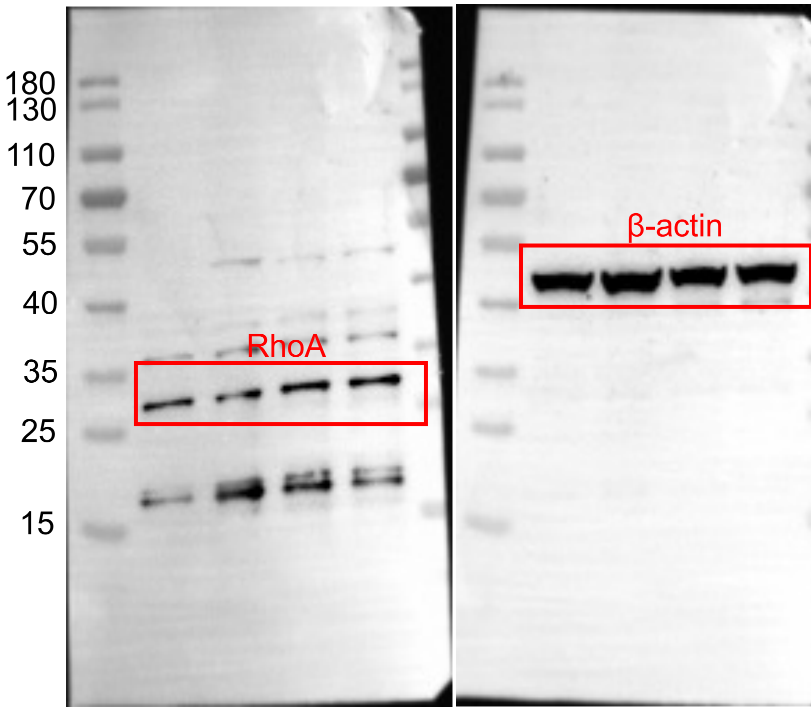

Figure 5H

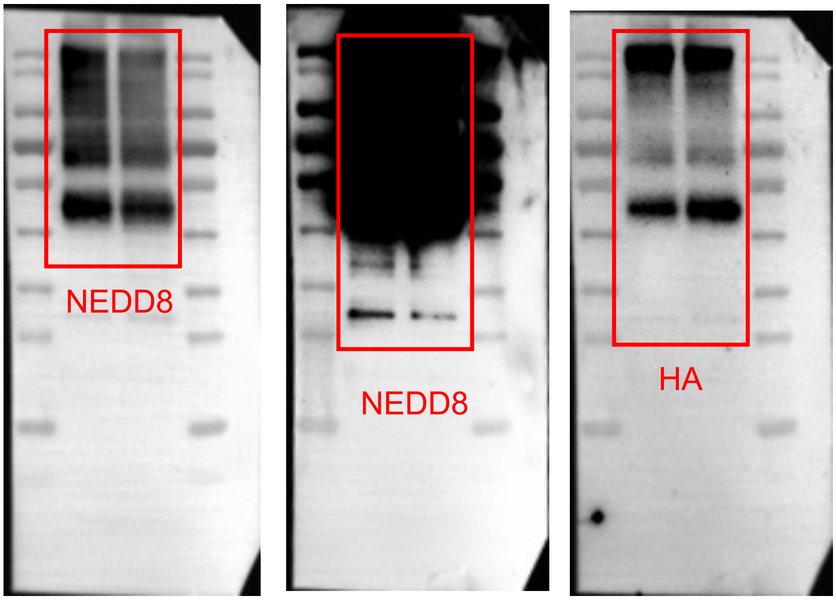

Figure 6C

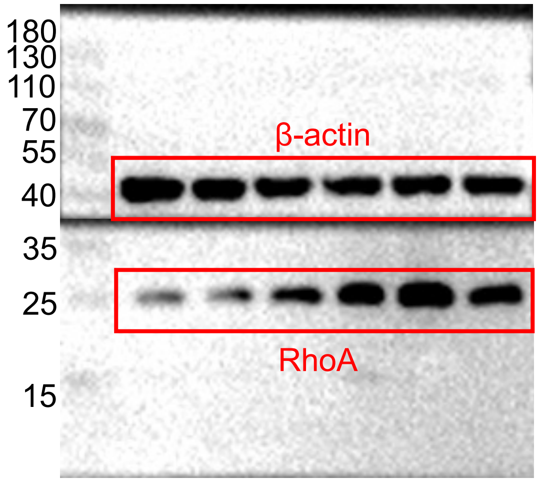

Figure 6D

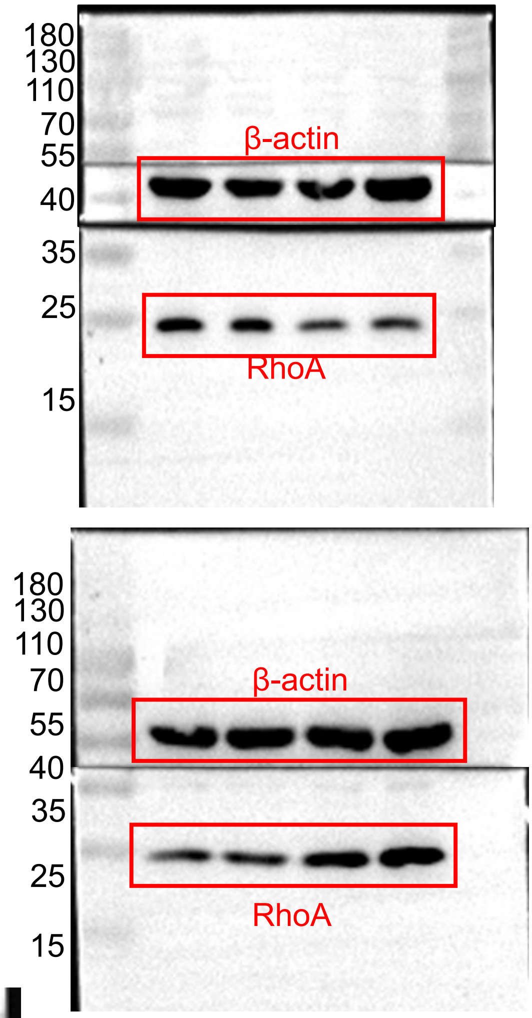

Figure 6E

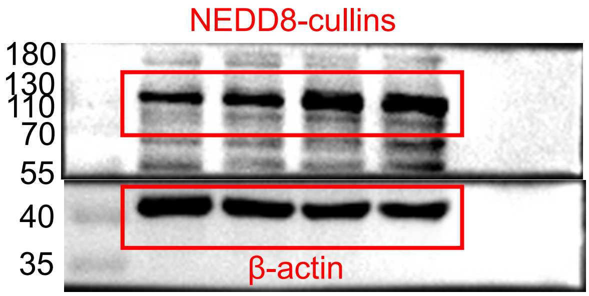

Figure 6G

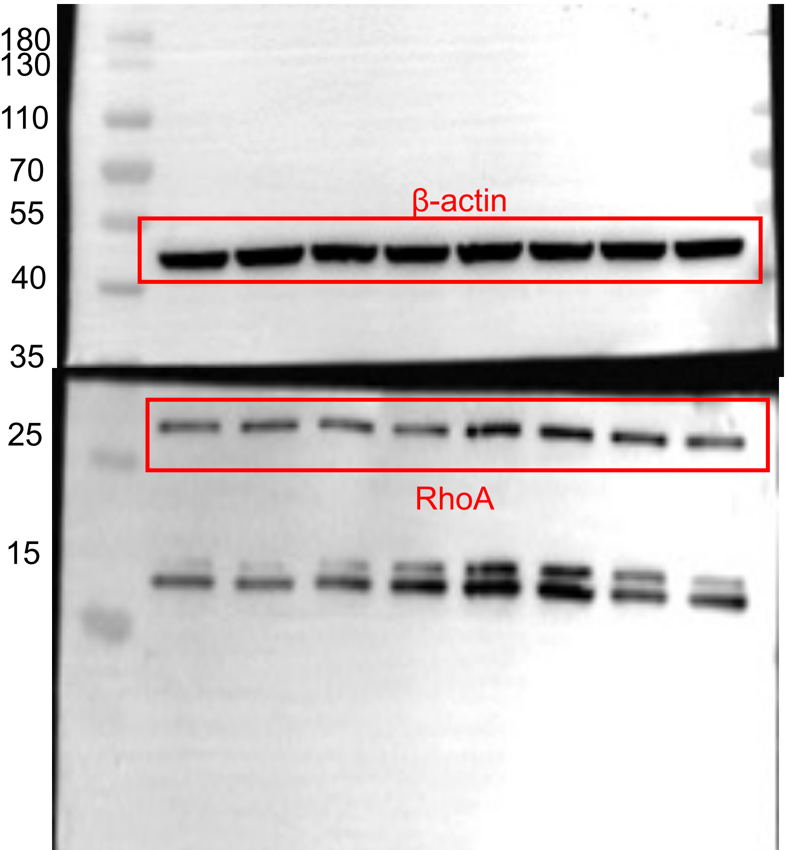

Figure 6H

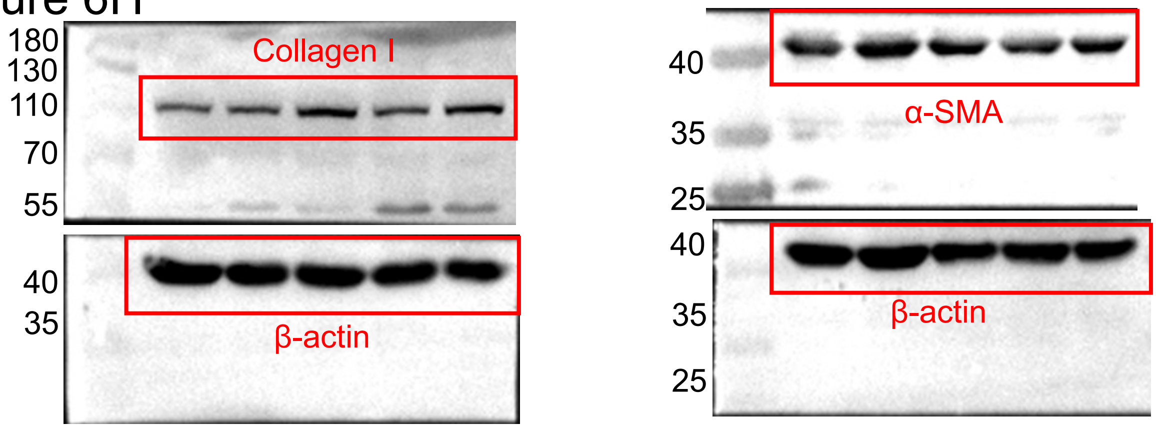
180
130
110
70
55
Collagen I
40
35
β-actin
40
35
25
α-SMA
40
35
25
β-actin

Figure 7C

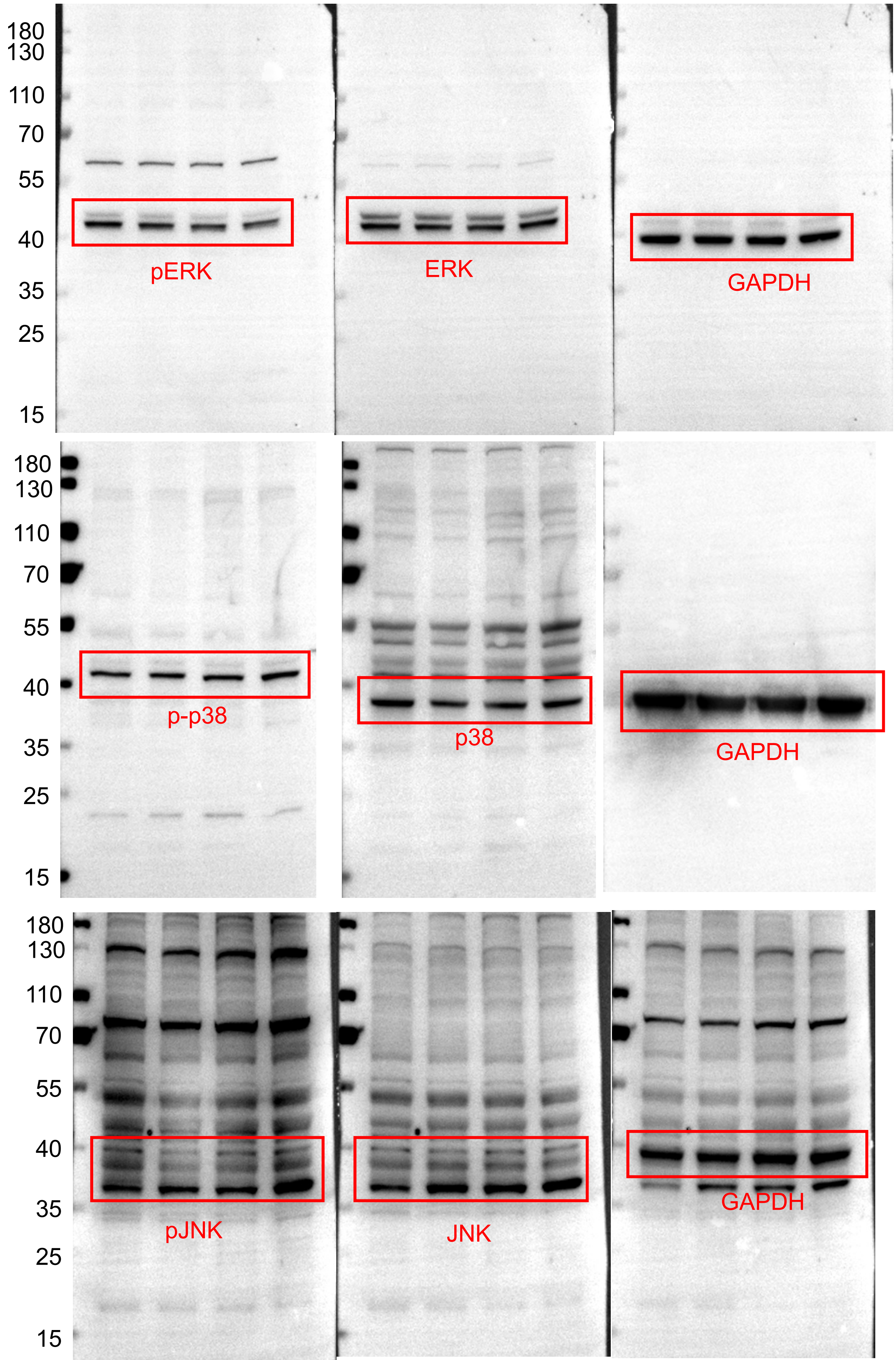
180
130
110
70
55
40
35
25
15
pERK
ERK
GAPDH
p-p38
p38
GAPDH
pJNK
JNK
GAPDH

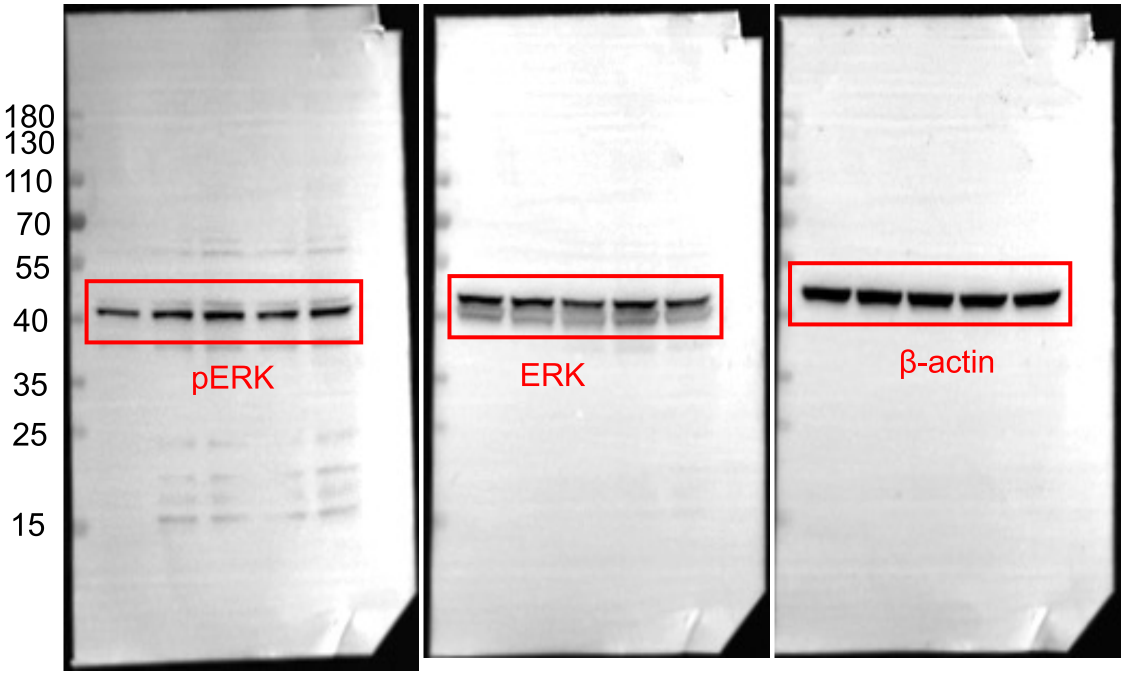
180
130
110
70
55
40
35
25
15
pERK
ERK
β-actin

Figure 8C

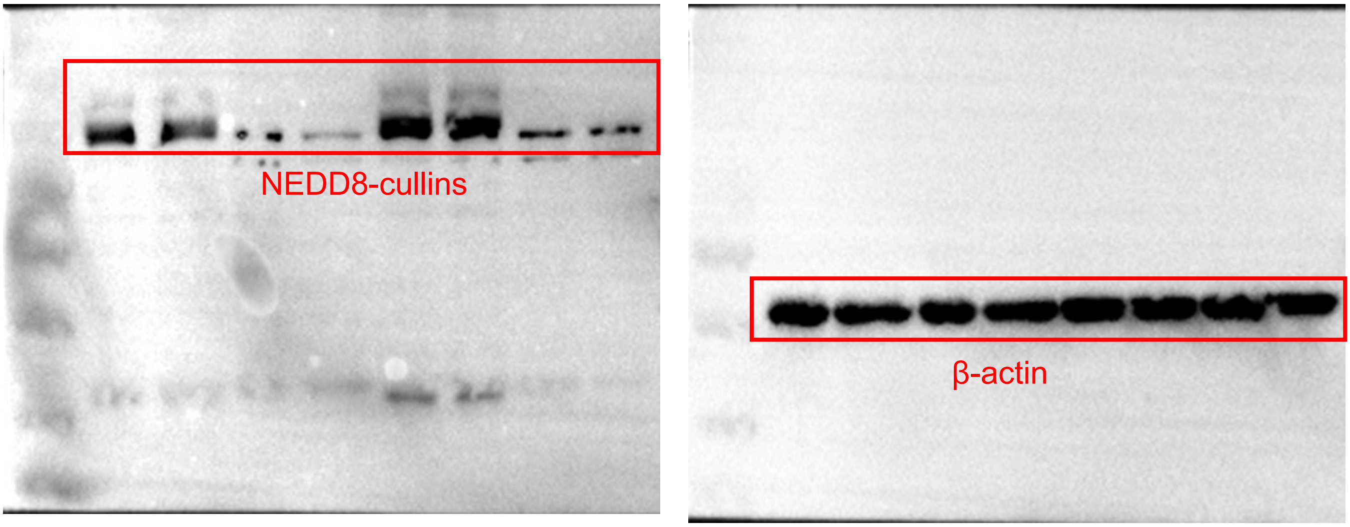

Figure 8H

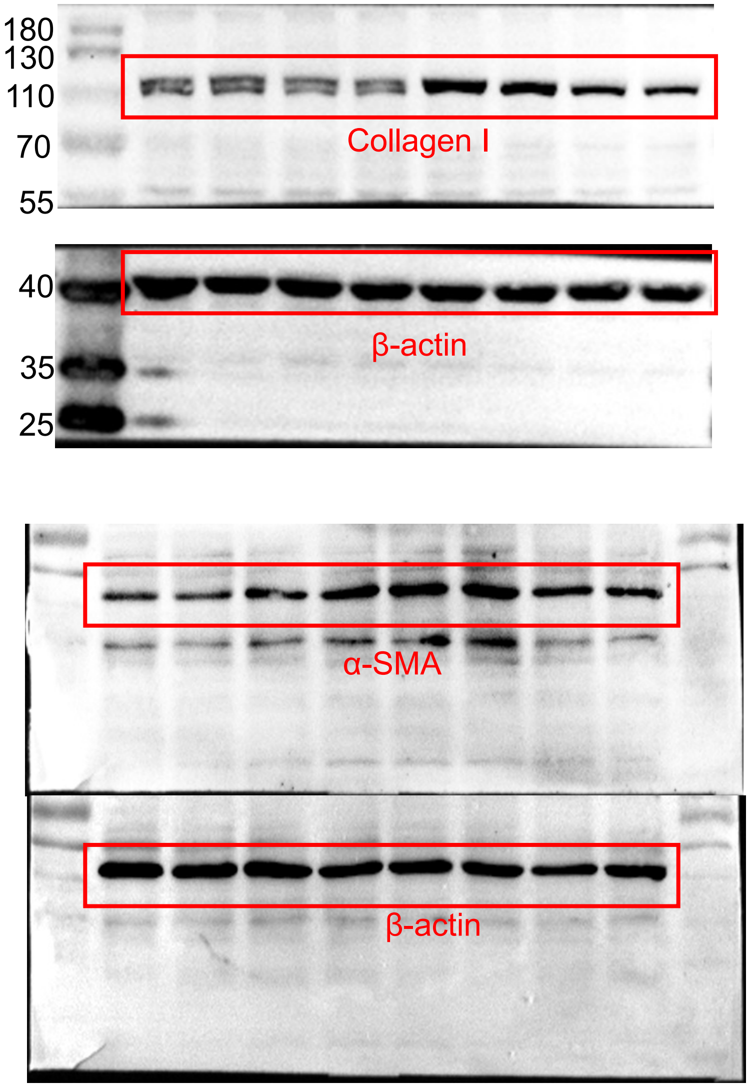

Supplement: Supplementary file 2 — Original WB gels [file 41401_2024_1460_MOESM2_ESM.pdf]
